# Dual-Band Impedance Matching Method for LWD Acoustic Transducers Considering Drill Collar Mechanical Coupling

**DOI:** 10.3390/mi17101133

**Published:** 2026-09-29

**Authors:** Xin Fu, Junyi Wu, Bei Tao, Qiangyu Han, Yang Gou

**Affiliations:** 1 College of Information Engineering, Shanghai Maritime University, Shanghai 201306, China; fuxin@shmtu.edu.cn (X.F.); 51he11o727@gmail.com (J.W.); 2 Shanghai Jian Qiao University, Shanghai 201306, China; 3 College of Logistic Engineering, Shanghai Maritime University, Shanghai 201306, China; h_qy021125@163.com; 4 Nantong Chaolin Intelligent Technology Co., Ltd., Nantong 226010, China

**Keywords:** LWD transducer, electrical impedance matching, electromechanical coupling, Gauss–Newton algorithm

## Abstract

The LWD acoustic transmitting system acts as a capacitive load with a narrow effective operating bandwidth and significant reactive power. In this paper, a KLM equivalent circuit model of the transmitting system, incorporating the mechanical coupling of the drill collar, is established based on its structural characteristics. This model is coupled with a dual-frequency impedance matching network to formulate an impedance function, whose parameters are solved using an adaptive Gauss–Newton algorithm. Subsequently, systematic electrical and acoustic tests were conducted on an anechoic water tank platform. Experimental results demonstrate that the active power at the transducer terminals is significantly enhanced within the target frequency bands after impedance matching. Taking 14 kHz and 4.5 kHz as examples, the peak-to-peak transmitting voltage at the transducer increases to approximately 1.75 times the pre-matching level at both frequencies. Meanwhile, the peak-to-peak receiving voltage of the hydrophone increases to about 1.7 and 3.5 times the pre-matching values at 14 kHz and 4.5 kHz, respectively. Furthermore, the transmitting voltage responses (TVR) of the quadrupole and monopole modes within their target bands improve by approximately 5–17 dB and 10–18 dB, respectively. Directivity measurements reveal that the horizontal main lobe of the quadrupole mode is highly pronounced, while the monopole mode maintains a near-circular radiation pattern with an overall increase in the sound pressure level.

## 1. Introduction

Logging While Drilling (LWD) technology enables real-time acquisition of geological and engineering parameters during drilling operations, significantly enhancing exploration success rates and drilling safety. It serves as core equipment for tackling complex hydrocarbon reservoirs [1,2,3]. Among these applications, LWD acoustic logging directly measures formation compressional and shear-wave velocities, which are critical for lithology identification, pore pressure prediction, and reservoir evaluation. The transmitter system of LWD acoustic instruments acts as the signal source, whose quality directly determines the reliability of logging data and the accuracy of extracted formation parameters, making it a key factor influencing log interpretation results. Poor signal quality can lead to difficulties in identifying formation characteristics, erroneous parameter calculations, and ultimately render the logging data unusable [4,5]. Sound waves are mechanical waves, and in practical applications, the admittance characteristics of transducers are significantly influenced by mechanical coupling with drill collars due to their tight integration during installation [6,7,8]. Currently, LWD acoustic logging primarily employs the monopole mode (12–15 kHz) to measure formation compressional wave velocity and the quadrupole mode (3–5 kHz) to measure shear wave velocity in soft formations. Conventional LWD transducers exhibit capacitive load behavior, and constrained by size limitations and technical development challenges, their admittance curves typically peak at frequencies above 20 kHz. Within the operational bands of both monopole and quadrupole modes, the transducers display pronounced reactive impedance components, which severely restrict active power delivery during excitation [9,10,11]. Therefore, a critical necessity arises to devise advanced dual-band impedance matching methodologies for LWD transducers, aiming to optimize acoustic emission efficiency and enhance measurement sensitivity via improved signal-to-noise ratio (SNR) [12,13].

Structurally, LWD transducers exhibit distinct differences from wireline logging tubular transducers, as their circumferential configuration is non-enclosed and positioned between the drill collar and outer protective shroud. This necessitates the development of robust electromechanical coupling models to accurately characterize the impedance properties of the emission system [14,15]. Furthermore, LWD transducers typically display multiple resonance peaks within their operational bandwidth, resulting in pronounced frequency-dependent impedance fluctuations that cannot be adequately represented by explicit analytical functions. Consequently, achieving dual-band impedance matching—simultaneously across the low-frequency quadrupole band (3–5 kHz) and high-frequency monopole band (12–15 kHz)—while maintaining sufficient bandwidth in both regimes, presents a significant technical challenge due to the inherently complex and non-uniform impedance behavior of the transducer [16,17,18].

Current research on impedance matching for LWD transducers remains largely fragmented. One line of research focuses on the encapsulation materials of the transducer, aiming to achieve acoustic matching for the piezoelectric ceramics by adjusting the ratio of epoxy resin to rubber [19,20]. Another line concentrates on electrical matching techniques. For instance, the Smith chart is often employed for impedance matching at a single frequency point [21,22]. Although real-frequency analysis based on the intrinsic impedance data of the transducer can facilitate a certain degree of broadband matching [23], this approach is primarily effective for large-scale underwater acoustic transducers, whose impedance frequency responses are relatively flat. It is, however, unsuitable for LWD transducers, which typically exhibit highly fluctuating impedance frequency responses [24]. Currently, most impedance matching methods in the LWD (logging while drilling) domain are confined to single-band impedance matching, and the fact that the impedance characteristics of the transducer change after it is mounted on the drill collar has not been thoroughly considered. Furthermore, certain logging instruments perform impedance matching at a single frequency point solely through a single inductor, thus lacking sufficient bandwidth characteristics [25,26]. Characterizing the impedance characteristics of transducers via equivalent circuit models is an effective approach for designing multi-band impedance matching networks. Currently, the Butterworth-Van Dyke (BVD) model is predominantly employed to represent LWD transducers. By leveraging impedance analysis data and nonlinear regression fitting algorithms, a BVD equivalent circuit model incorporating multiple resonances can be successfully derived. However, once installed on a drill collar, the transducer is subjected to confining pressure from both the outer cover plate and the inner drill collar, effectively forming a composite structure [27,28,29,30,31,32,33]. Consequently, relying on the equivalent circuit model under free-boundary conditions makes it difficult to accurately calculate the parameters for the impedance matching network.

To address the aforementioned issues, this paper further incorporates the practical coupling between the LWD transducer and the drill collar to construct a Krimholtz–Leedom–Matthaei (KLM) model for the LWD instrument transmitting system. A dual-band impedance matching network is integrated, and its parameters are optimized by an adaptive Gauss–Newton algorithm. This hybrid approach effectively overcomes the limitation of conventional single algorithms, which are prone to falling into local optima. Finally, water tank experiments were conducted to validate the proposed method. The experimental results demonstrate that, after impedance matching, the active power of the LWD transmitting system increases within both target frequency bands. Concurrently, the transmitting voltage response (TVR) improves by 15 and 17 dB.

## 2. Theoretical Derivation of the Electromechanical Equivalent Circuit Model for the Drill Collar–Transducer Structure

**Figure 1:**
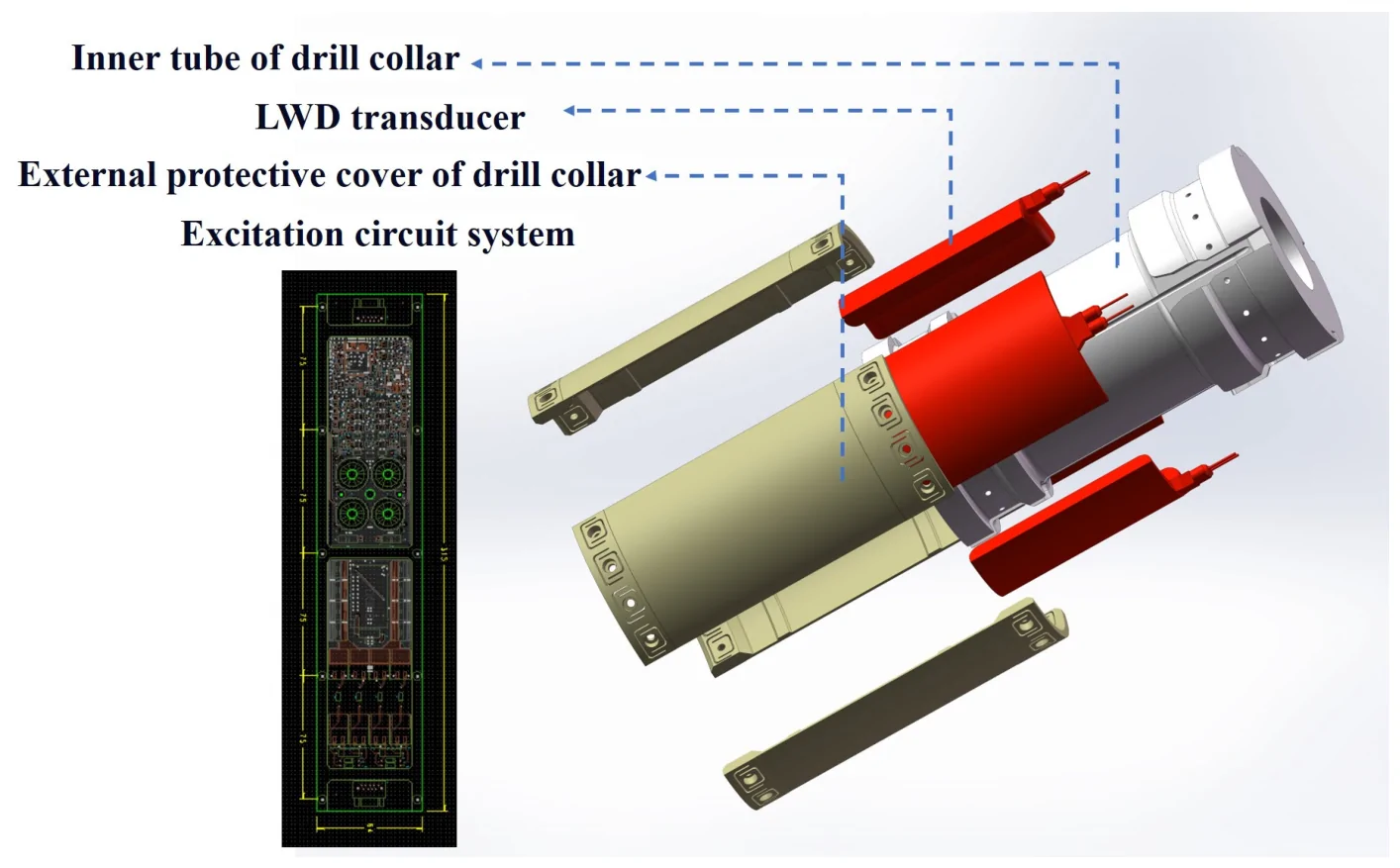
Structure of the LWD instrument transmitting system.

As illustrated in Figure 1, the transmitter sub of the LWD tool primarily consists of an LWD transducer, an inner mandrel, an outer protective cover, and the transmitter circuitry. While the transducer remains free from confining pressure in a free state, its installation on the drill collar subjects it to confining pressures from both the inner mandrel and the outer cover. This mechanical loading alters the intrinsic impedance characteristics of the transducer, thereby degrading the performance of the impedance matching network originally designed for the free state. Consequently, it is necessary to develop a novel equivalent circuit model tailored to the specific features of the LWD transmitter system. By incorporating the mechanical parameters of the drill collar, this approach aims to enhance the electromechanical integration of the overall system.

**Figure 2:**
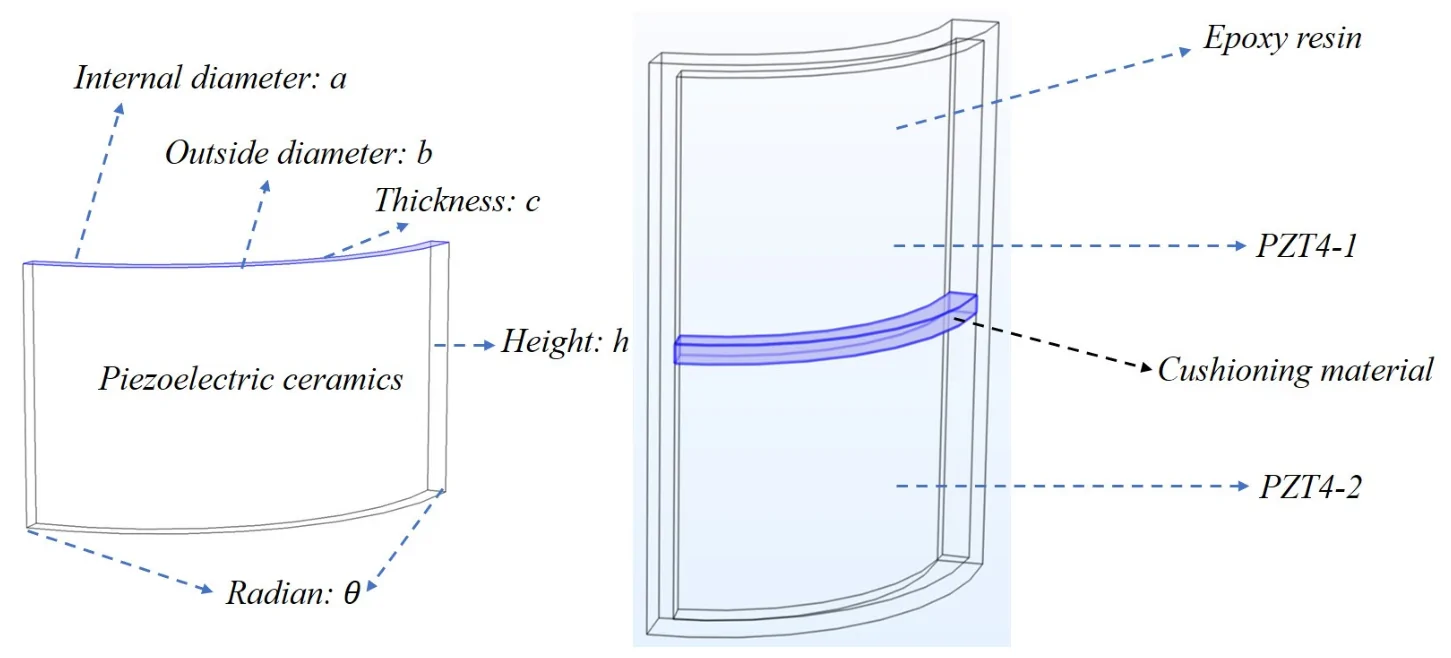
3D assembly diagram of the LWD transducer.

Figure 2 illustrates the 3D schematic of the LWD transducer. It primarily consists of two piezoelectric ceramics, a central filler material, and an epoxy resin encapsulation. The inner diameter, outer diameter, thickness, and height of the piezoelectric ceramics are denoted as *a*, *b*, *c*, and *h*, respectively, with the arc angle θ typically set at 89°. The piezoelectric ceramic material used in this study is PZT-4, and the central buffering material is aluminum. Once the transducer is mounted on the drill collar, the subsequent KLM model parameters will be directly related to the materials and dimensions of both the transducer and the drill collar.

**Figure 3:**
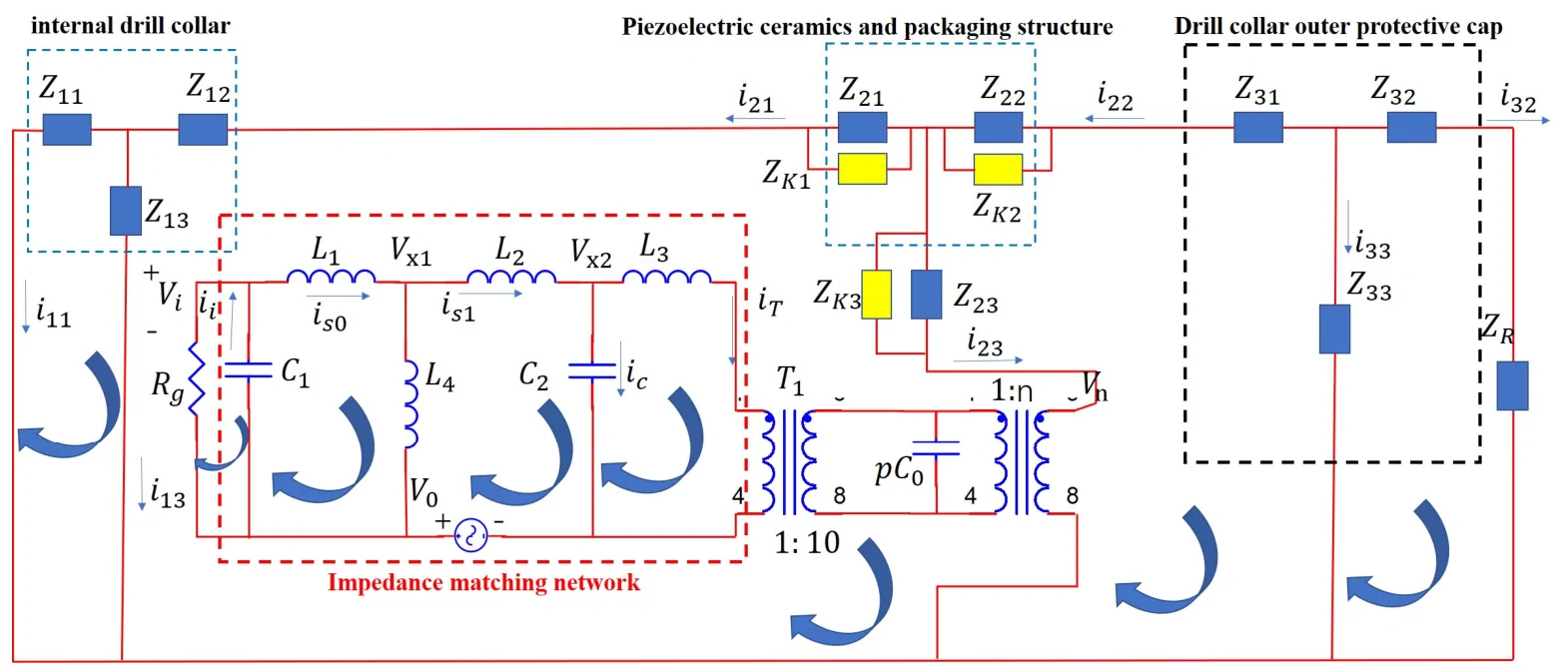
KLM model of the improved LWD transducer.

Upon installation of the LWD transducer onto the drill collar transmitter sub, the inner collar, transducer, and outer collar protective cap collectively form a typical composite structure. Consequently, Figure 3 illustrates the improved KLM equivalent circuit for the proposed LWD transmitting system. In this model, $Z_{11}$, $Z_{12}$, and $Z_{13}$ represent the equivalent impedances of the inner collar, while $Z_{21}$, $Z_{22}$, and $Z_{23}$ denote the equivalent impedances of the piezoelectric ceramic section. Since the piezoelectric ceramics are embedded within the epoxy resin and separated by buffering materials, the impedance of this section is formed by the parallel connection of $Z_{K1}$, $Z_{K2}$, and $Z_{K3}$. Serving as the outermost layer in direct contact with the formation, the drill collar protective cap acts as the initiation point for acoustic signal radiation; its corresponding impedances are designated as $Z_{31}$, $Z_{32}$, and $Z_{33}$. Furthermore, $Z_{R}$ represents the confining pressure surrounding the entire system. Outside the static capacitance $C_{0}$ of the transducer lies the dual-band impedance matching network utilized in this study, which comprises a Π -type network and a T-type network connected in series. Subsequent parameter calculations for this impedance matching network will be conducted based on the aforementioned KLM model.

The mechanical impedances $Z_{ij}$ (*i* = 1, 2, 3; *j* = 1, 2, 3) in Figure 3 represent the equivalent impedances of the inner drill collar [26], which is modeled as a thick-walled cylindrical shell undergoing axisymmetric radial vibration. Based on the wave equation in cylindrical coordinates, the radial displacement field is expressed in terms of Bessel functions $J_{1}(\tilde{k}r)$ and $Y_{1}(\tilde{k}r)$ of order one, where $\tilde{k}=\omega /C_{L}$ is the longitudinal wavenumber with $C_{L}=\sqrt{E/[\rho (1-u^{2})]}$ being the longitudinal wave speed in the collar material. The impedance matrix entries in Equations (1)–(3) are derived by evaluating the force–velocity relations at the inner (r=a) and outer (r=b) cylindrical boundaries of the drill collar tube.

where

(1) $$Z_{11}=\frac{4h\rho \sqrt{E{\left(\rho (1-\mu^{2})\right)}^{-1}}}{\tilde{k}[J1\left(\tilde{k}R2\right)Y1\left(\tilde{k}R1\right)-J1\left(\tilde{k}R1\right)Y1\left(\tilde{k}R2\right)]}\left[\frac{J1\left(\tilde{k}R2\right)Y0\left(\tilde{k}R1\right)-J0\left(\tilde{k}R1\right)Y1\left(\tilde{k}R2\right)}{J1\left(\tilde{k}R1\right)Y0\left(\tilde{k}R1\right)-J0\left(\tilde{k}R1\right)Y1\left(\tilde{k}R1\right)}\right]\;-j\frac{4h\rho \sqrt{E{\left(\rho \left(1-\mu^{2}\right)\right)}^{-1}}(1-\mu )}{R1{\tilde{k}}^{2}[J1\left(\tilde{k}R1\right)Y0\left(\tilde{k}R1\right)-J0\left(\tilde{k}R1\right)Y1(\tilde{k}R1)]}$$

(2) $$Z_{22}=\frac{4h\rho \sqrt{E{\left(\rho (1-\mu^{2})\right)}^{-1}}}{\tilde{k}[J1\left(\tilde{k}R2\right)Y1\left(\tilde{k}R1\right)-J1\left(\tilde{k}R1\right)Y1\left(\tilde{k}R2\right)]}\left[\frac{J1\left(\tilde{k}R1\right)Y0\left(\tilde{k}R2\right)-J0\left(\tilde{k}R2\right)Y1\left(\tilde{k}R1\right)}{J1\left(\tilde{k}R2\right)Y0\left(\tilde{k}R2\right)-J0\left(\tilde{k}R2\right)Y1\left(\tilde{k}R2\right)}\right]\;+j\frac{4h\rho \sqrt{E{\left(\rho \left(1-\mu^{2}\right)\right)}^{-1}}(1-\mu )}{R2\pi {k\tilde{k}}^{2}[J1\left(\tilde{k}R2\right)Y0\left(\tilde{k}R2\right)-J0\left(\tilde{k}R2\right)Y1(\tilde{k}R2)]}$$

(3) $$Z_{13}=j\frac{4h\rho \sqrt{E{\left(\rho (1-\mu^{2})\right)}^{-1}}}{\tilde{k}[J1\left(\tilde{k}R2\right)Y1\left(\tilde{k}R1\right)-J1\left(\tilde{k}R1\right)Y1\left(\tilde{k}R2\right)]}$$

In Equations (1)–(3), ρ is the density of the inner tube of drill collar, E is the Young’s modulus of the inner tube of drill collar, μ is the Poisson’s ratio, h represents the height of the drill collar sub. $R_{1}$ and $R_{2}$ represent the inner diameter and outer diameter of the inner tube, respectively *J*($\tilde{k}$*r*) and *Y*($\tilde{k}$*r*) are the first and second kind of Bessel functions of order one. Dimensionally, each $Z_{ij}$ has units of mechanical impedance (kg/s, or equivalently Ns/m, as ρ has units of kg/(m²·s), the geometric factor *h* has units of m, and the products of Bessel functions in the numerator and denominator are dimensionless. The transformer in Figure 3 represents the coupling relationship only; the actual current flows through it.

Equations (4)–(7) describe the equivalent mechanical impedances of the piezoelectric ceramic stack. Unlike a purely elastic medium, the piezoelectric material exhibits electromechanical coupling, characterized by the piezoelectric strain constant $d_{33}$ and the difference between the short-circuit elastic compliance $s_{33}^{E}$. The effective Young’s modulus for the piezoceramic under the electrical boundary conditions of the KLM model is given by $E=1/s_{33}^{E}$, and the corresponding longitudinal wave speed is c=E/ρ. The expressions in Equations (4)–(7) follow the same cylindrical-shell impedance formulation as Equations (1)–(3), with the geometric parameters now referring to the piezoceramic ring. The transformer turns ratio n in Figure 3 which couples the electrical and mechanical domains, is proportional to $d_{33}$ and to the ration $h/s_{11}^{E}\varepsilon_{33}^{T}$.

(4) $$Z_{21}\frac{\pi^{3}{\left(\tilde{k}R4\right)}^{2}\rho_{0}\sqrt{E_{0}{\left(\rho_{0}(1-{\mu_{0}}^{2})\right)}^{-1}}R3h}{2j}\left[\frac{Y1\left(\tilde{k}R4\right)J0\left(\tilde{k}R3\right)-J1\left(\tilde{k}R4\right)Y0\left(\tilde{k}R3\right)}{J1\left(\tilde{k}R4\right)Y1\left(\tilde{k}R3\right)-J1\left(\tilde{k}R3\right)Y1\left(\tilde{k}R4\right)}\;+\frac{s_{11}^{E}+s_{12}^{E}}{s_{11}^{E}\tilde{k}R4}\right]-j\frac{{{h_{0}R3\rho }_{0}\sqrt{E_{0}{\left(\rho_{0}(1-\mu^{2})\right)}^{-1}}\pi }^{2}\tilde{k}R4}{J1\left(\tilde{k}R4\right)Y1\left(\tilde{k}R3\right)-J1\left(\tilde{k}R3\right)Y1\left(\tilde{k}R4\right)}$$

(5) $$Z_{22}\frac{\pi^{3}{\left(\tilde{k}R3\right)}^{2}\rho_{0}\sqrt{E_{0}{\left(\rho_{0}(1-{\mu_{0}}^{2})\right)}^{-1}}R4h_{0}}{2j}\left[\frac{Y1\left(\tilde{k}R4\right)J0\left(\tilde{k}R3\right)-J1\left(\tilde{k}R4\right)Y0\left(\tilde{k}R3\right)}{J1\left(\tilde{k}R4\right)Y1\left(\tilde{k}R3\right)-J1\left(\tilde{k}R3\right)Y1\left(\tilde{k}R4\right)}+\;\frac{s_{11}^{E}+s_{12}^{E}}{s_{11}^{E}\tilde{k}R3}\right]-j\frac{{{h_{0}R4\rho }_{0}\sqrt{E_{0}{\left(\rho_{0}(1-{\mu_{0}}^{2})\right)}^{-1}}\pi }^{2}\tilde{k}R3}{J1\left(\tilde{k}R4\right)Y1\left(\tilde{k}R3\right)-J1\left(\tilde{k}R3\right)Y1\left(\tilde{k}R4\right)}$$

(6) $$Z_{33}=j\frac{\pi^{2}R4R3\tilde{k}h_{0}\rho_{0}\sqrt{E_{0}{\left(\rho_{0}(1-{\mu_{0}}^{2})\right)}^{-1}}}{J1\left(\tilde{k}R4\right)Y1\left(\tilde{k}R3\right)-J1\left(\tilde{k}R3\right)Y1\left(\tilde{k}R4\right)}$$

(7) $$C_{0}=\frac{2\pi pR4h_{0}\varepsilon_{33}^{T}S_{33}^{D}}{S_{33}^{E}},\;n=\frac{2\pi R3{h_{0}d}_{33}}{S_{33}^{E}}$$

In Equations (4)–(7), $\rho_{0}$, $E_{0}$, $R_{3}$, $R_{4}$, $h_{0}$, $\varepsilon_{33}^{T}$, $d_{33}$, $\mu_{1}$, $S_{33}^{E}$ and $S_{33}^{D}$ are the density of piezoelectric ceramic materials, Young’s modulus of piezoelectric ceramic materials, Inner diameter of piezoelectric ceramic materials, Outer diameter of piezoelectric ceramic materials, height of piezoelectric ceramic materials, free permittivity, piezoelectric strain constant, Poisson’s ratio, short-circuit elastic compliance, open-circuit elastic compliance, respectively.

Equations (8)–(11) give the mechanical impedances of the outer drill collar protective cover. This component serves as the outermost mechanical layer in direct contact with the borehole fluid, and thus acts as the acoustic radiation interface. Its impedance formulation is structurally identical to that of the inner collar (Equations (1)–(3)), with material and geometric parameters (ρ,E,u,a,b,h) now specific to the protective cover.

(8) $$\begin{array}{c} Z_{31}\\ =j\frac{4h_{1}\rho_{1}\sqrt{E_{1}{\left(\rho_{1}(1-{\mu_{1}}^{2})\right)}^{-1}}}{\tilde{k}[J1\left(\tilde{k}R6\right)Y1\left(\tilde{k}R5\right)-J1\left(\tilde{k}R5\right)Y1\left(\tilde{k}R6\right)]}\left[\frac{J1\left(\tilde{k}R6\right)Y0\left(\tilde{k}R5\right)-J0\left(\tilde{k}R5\right)Y1\left(\tilde{k}R6\right)}{J1\left(\tilde{k}R5\right)Y0\left(\tilde{k}R5\right)-J0\left(\tilde{k}R5\right)Y1\left(\tilde{k}R5\right)}\right]\\ -j\frac{4h_{1}\rho_{1}\sqrt{E_{1}{\left(\rho_{1}\left(1-{\mu_{1}}^{2}\right)\right)}^{-1}}(1-\mu_{1})}{R5{\tilde{k}}^{2}[J1\left(\tilde{k}R5\right)Y0\left(\tilde{k}R5\right)-J0\left(\tilde{k}R5\right)Y1(\tilde{k}R5)]} \end{array}$$

(9) $$Z_{32}=\frac{4h_{1}\rho_{1}\sqrt{E_{1}{\left(\rho_{1}(1-{\mu_{1}}^{2})\right)}^{-1}}}{\tilde{k}[J1\left(\tilde{k}R6\right)Y1\left(\tilde{k}R5\right)-J1\left(\tilde{k}R5\right)Y1\left(\tilde{k}R6\right)]}\left[\frac{J1\left(\tilde{k}R5\right)Y0\left(\tilde{k}R6\right)-J0\left(\tilde{k}R6\right)Y1\left(\tilde{k}R5\right)}{J1\left(\tilde{k}R6\right)Y0\left(\tilde{k}R6\right)-J0\left(\tilde{k}R6\right)Y1\left(\tilde{k}R6\right)}\right]\;+j\frac{4h_{1}\rho_{1}\sqrt{E_{1}{\left(\rho_{1}\left(1-{\mu_{1}}^{2}\right)\right)}^{-1}}(1-\mu_{1})}{R6\pi {\tilde{k}}^{2}[J1\left(\tilde{k}R6\right)Y0\left(\tilde{k}R6\right)-J0\left(\tilde{k}R6\right)Y1(\tilde{k}R6)]}$$

(10) $$Z_{33}=j\frac{4h_{1}\rho_{1}\sqrt{E_{1}{\left(\rho_{1}(1-{\mu_{1}}^{2})\right)}^{-1}}}{k[J1\left(kR6\right)Y1\left(kR5\right)-J1\left(kR5\right)Y1\left(kR6\right)]}$$

(11) $$Z_{R}=\rho_{w}C_{w}S_{1}[1-\frac{J_{1}(2k_{w}a)}{k_{w}a}+\frac{jS_{1}(2k_{w}a)}{k_{w}a}]$$

In Equations (8)–(11), $\rho_{1}$, $E_{1}$, $R_{5}$, $R_{6}$, $h_{1}$, $\mu_{1}$ are the density of drill collar protector, Young’s modulus of drill collar protector, Inner diameter of drill collar protector, Outer diameter of drill collar protector, height of drill collar protector, free permittivity, piezoelectric strain constant, Poisson’s ratio, short-circuit elastic compliance, open-circuit elastic compliance, respectively. Here, $Z_{R}$ is the LWD transmitter system load impedance, where $\rho_{w}$, $C_{w}$, and $k_{w}$ are the density, sound velocity, and wave number of water, respectively, while *a* denotes the radius of the radiation surface.

In Equation (12), $\rho_{s}$, $C_{ss}$, $h_{2}$ are the density of cushioning material, sound velocity of cushioning material, height of cushioning material, respectively.

(12) $$Z_{k1}=Z_{k2}=\frac{2\pi R4h_{2}\rho_{s}C_{ss}}{j}c\mathrm{os}\left(\frac{k_{p}(R4-R3)}{4}\right),\;Z_{k3}=\frac{{j2\pi R3h_{2}\rho }_{s}C_{ss}}{sin(k_{p}(R4-R3))}$$

Establish the Kirchhoff network equation based on the equivalent circuit in Figure 3:

(13) $${(Z}_{32}+Z_{R})i_{32}=Z_{33}i_{33}$$

(14) $${(Z}_{31}+\frac{Z_{22}Z_{k2}}{Z_{22}+Z_{k2}})i_{22}+\frac{Z_{23}Z_{k3}}{Z_{23}+Z_{k3}}i_{23}+V_{n}=Z_{33}i_{33}$$

(15) $$\left(\frac{Z_{21}Z_{k1}}{Z_{21}+Z_{k1}}+Z_{12}\right)i_{21}{+Z_{13}i}_{13}=V_{n}{+\frac{Z_{23}Z_{k3}}{Z_{23}+Z_{k3}}i}_{23}$$

(16) $$Z_{11}i_{11}=Z_{13}i_{13},i_{13}+i_{11}=i_{21},i_{21}+i_{23}=i_{22},i_{22}+i_{32}=i_{33}$$

From Formulas (15) and (16):

(17) $$A_{1}\left(i_{32}+i_{33}\right)=V_{n}+A_{2}i_{23},i_{23}=\frac{V_{n}}{\rho C_{0}}$$

Combining Formulas (13)–(16) and (17), it can be obtained:

(18) $$i_{32}=\frac{V_{n}+\frac{Z_{23}Z_{k3}}{Z_{23}+Z_{k3}}i_{23}}{A_{3}-A_{4}},i_{33}=A_{5}\frac{V_{n}+\frac{Z_{23}Z_{k3}}{Z_{23}+Z_{k3}}i_{23}}{A_{3}-A_{4}}$$

Substitute Formula (18) into Formula (17):

(19) $$Z_{T}=\frac{A_{1}+A_{1}A_{5}-A_{3}+A_{4}}{A_{3}-A_{4}-\frac{Z_{23}Z_{k3}}{Z_{23}+Z_{k3}}A_{1}(1+A_{5})}$$

where

(20) $$A_{1}=-\left(\frac{Z_{21}Z_{k1}}{Z_{21}+Z_{k1}}+Z_{12}+\frac{Z_{11}Z_{13}}{Z_{11}+Z_{13}}\right),A_{2}=\left(\frac{Z_{21}Z_{k1}}{Z_{21}+Z_{k1}}+Z_{12}+\frac{Z_{23}Z_{k3}}{Z_{23}+Z_{k3}}\right)$$

(21) $$A_{3}=Z_{33}(Z_{32}+Z_{R}),A_{4}=(Z_{31}+\frac{Z_{22}Z_{k2}}{Z_{22}+Z_{k2}})(Z_{33}+Z_{32}+Z_{R})$$

(22) $$A_{5}=\frac{Z_{R}Z_{32}^{2}}{Z_{31}Z_{33}}$$

The parameters of the broadband impedance matching network were calculated based on this model.

## 3. Calculation of Dual-Frequency Impedance Matching Network Parameters Considering the Influence of Drill Collar Coupling

The calculation of impedance matching network parameters requires the actual parameters of the transducer. However, once the transducer is mounted on the drill collar, its impedance characteristics change due to the mechanical pressure exerted by the inner mandrel and the protective cap. Conventional impedance matching relies on data measured in a free state at the factory, which inevitably introduces significant errors into the parameter calculation. To address this issue, this study fully accounts for the mechanical coupling characteristics of the drill collar by establishing an equivalent circuit model for the entire transmitter system. Consequently, the actual impedance function of the transducer under drill collar installation conditions is derived. The material parameters of the transducer are shown in Table 1. Table 2 presents the dimensional parameters of the transducer.

**Figure 4:**
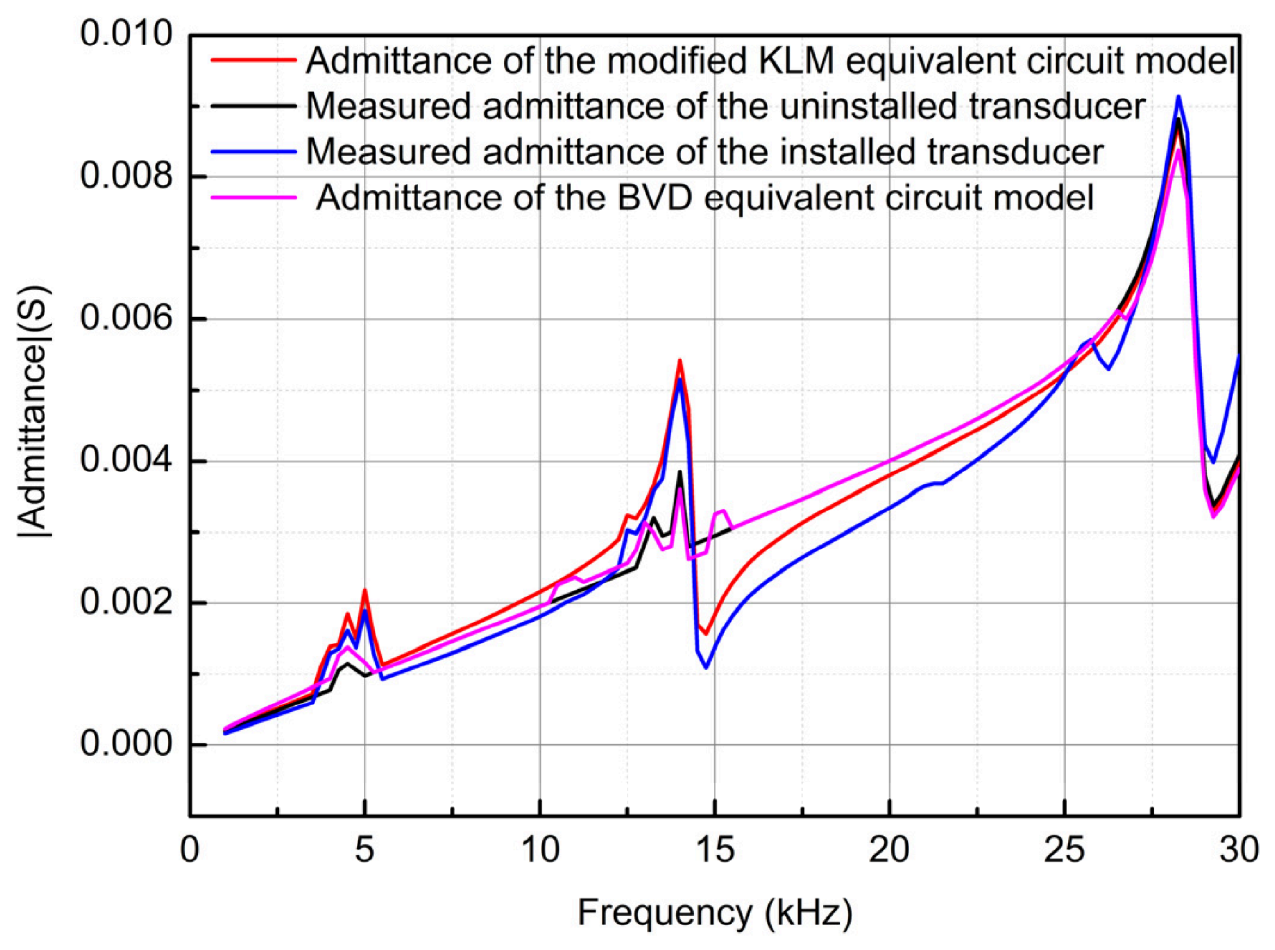
Consider the equivalent circuit model of the transducer KLM for the mechanical coupling characteristics of the drill collar.

As illustrated in Figure 4, a comparison is presented between the measured admittance curves of the transducer with and without installation on the drill collar, alongside the admittance curve derived from the proposed equivalent circuit. It can be observed that the admittance characteristics of the transducer alter significantly upon installation. Noticeable deviations exist between the admittance curves before and after installation, particularly within the two resonant frequency bands. Meanwhile, the admittance curve of the BVD equivalent model fails to capture the admittance characteristics of the transducer after it is mounted on the drill collar. Moreover, even when compared to the unmounted state, the model does not adequately represent the admittance characteristics of the transducer at the resonance frequency. However, the equivalent circuit model proposed in this study, which incorporates the mechanical coupling characteristics of the drill collar, accurately characterizes the impedance behavior of the entire transmitter system with the transducer integrated into the instrument. Consequently, utilizing this equivalent circuit model facilitates the accurate calculation of optimal impedance matching network parameters.

Based on the impedance distribution shown in Figure 3, the impedance function of the model system was derived using Kirchhoff’s voltage and current laws:

(23) $$\{\begin{array}{c} V_{0}+i_{i}R_{g}=\frac{\left(i_{s0}-i_{i}\right)}{j\omega C_{1}}\;\\ j\omega L_{1}i_{12}+j\omega L_{4}(i_{s1}-i_{s0})=\frac{\left(i_{s0}-i_{i}\right)}{j\omega C_{1}}\\ j\omega L_{2}i_{s1}+\frac{i_{c}}{j\omega C_{2}}=j\omega L_{4}\left(i_{s1}-i_{s0}\right)\\ j\omega L_{1}i_{12}+j\omega L_{4}\left(i_{s1}-i_{s0}\right)=\frac{\left(i_{s0}-i_{i}\right)}{j\omega C_{1}}\\ j\omega L_{2}i_{s1}+\frac{i_{c}}{j\omega C_{2}}=j\omega L_{4}(i_{s1}-i_{s0}) \end{array}$$

(24) $$\{\begin{array}{c} i_{T}\left(j\omega L_{3}+Z_{T}\right)=\frac{i_{c}}{j\omega C_{2}}\\ j\omega \left(L_{1}i_{s0}+L_{4}i_{s1}+L_{3}i_{T}\right)+\frac{Z_{T}i_{T}}{n^{2}}=V_{i}+2R_{g}i_{i}\\ V_{x1}=V_{i}-j\omega L_{1}i_{i}-\omega^{2}V_{i}C_{1}L_{1}\\ V_{x2}=i_{s1}j\omega C_{2}+V_{x1} \end{array}$$

(25) $$i_{s0}=i_{i}-V_{i}j\omega C_{1}$$

(26) $$i_{s1}=i_{i}-j\omega \left(V_{i}C_{1}-V_{x1}L_{4}\right)$$

(27) $$i_{T}=i_{s1}-j\omega C_{2}V_{x2}$$

By solving Equations (23)–(27) simultaneously, the impedance function of the entire equivalent circuit can be obtained.

(28) $$Z_{s}=\frac{V_{i}}{i_{i}}=\frac{\left(K_{18}K_{16}-K_{17}K_{15})+j(K_{17}K_{16}+K_{18}K_{15}\right)}{-(K_{15}^{2}+K_{16}^{2})}$$

(29) Re[Zs]=K17K15−K18K16(K152+K162)

(30) Im[Zs]=K17K15+K18K16−(K152+K162)

The coefficients $K_{i}$ in Equations (28)–(30) serve as notational shorthand; they are algebraic groupings of the matching network components. Their full expressions are given in Appendix A. They do not introduce any new physical parameters beyond those already defined in the KLM equivalent circuit. Specifically, all $K_{i}$ are polynomial combinations of the following known quantities:

The six lumped components of the dual-band matching network: $C_{1}$, $C_{2}$, $L_{1}$, $L_{2}$, $L_{3}$, $L_{4}$.

The angular frequency ω=2πf.

The grouping of these coefficients follows a natural hierarchy dictated by the network topology:

Coefficients $K_{1}-K_{6}$ originate from the $R_{g}$ Π-type input stage of the matching network and represent the algebraic contributions of $L_{1},L_{4},C_{1}$.

Coefficients $K_{7}-K_{14}$ arise from the T-type output stage and encapsulate the coupling effects of $L_{1},L_{4},C_{1}$.

Coefficients $K_{15}-K_{18}$ emerge from the cross-coupling terms between the Π and T stages when the full network impedance.

All $K_{i}$ are rational functions of ω (i.e., ratios of polynomials in ω), which guarantees smooth frequency dependence. Their complete explicit forms are provided in Appendix A.

Subsequently, the parameters of the impedance matching network are determined via an adaptive Gauss–Newton algorithm based on this impedance function.

Based on the input impedance model of the aforementioned impedance matching network, the parameter vector to be optimized is defined as:

(31) $$\theta ={\left[\begin{array}{ccccc} C_{1} & L_{1} & L_{4} & L_{2} & C_{2\;}\;\;\;L_{3} \end{array}\right]}^{T}$$

The input impedance of the matched system is defined as:

(32) $$Z_{in}\left(f,\theta \right)=R_{in}\left(f,\theta \right)+jX_{in}\left(f,\theta \right)$$

The reference impedance is set to $R_{g}$, and the target frequency band is defined as F=[3 kHz,5 kHz]∪[12 kHz,15 kHz]. The goal of impedance matching is to make the real part of the input impedance as close to the reference impedance $R_{g}$ as possible within the target band, while driving the imaginary part toward zero. To achieve this, a weighted nonlinear least-squares objective function is formulated as follows:

(33) $$E\left(\theta \right)=\sum_{f_{k}\in F}\left[w_{R}(f_{k})(R_{in}(f_{k},\theta )-R_{g}{)}^{2}+w_{X}(f_{k})X_{in}^{2}(f_{k},\theta )\right]$$

where $w_{R}\left(f_{k}\right)$ and $w_{X}\left(f_{k}\right)$ represent the weights of the real and imaginary error terms at frequency point $f_{k}$, respectively.

To enable parameter updates via the Gauss–Newton method, we stack the real and imaginary deviations in the target frequency band in the order of frequency points, thereby constructing a real residual vector.

(34) $$e(\theta )=\left[\begin{array}{c} \sqrt{w_{R}(f_{1})}(R_{in}(f_{1},\theta )-R_{g})\\ \sqrt{w_{X}(f_{1})}X_{in}(f_{1},\theta )\\ \vdots \\ \sqrt{w_{R}(f_{N})}(R_{in}(f_{N},\theta )-R_{g})\\ \sqrt{w_{X}(f_{N})}X_{in}(f_{N},\theta ) \end{array}\right]$$

The normalized impedance deviation for the k iteration is defined as:

(35) $$X\left(k\right)=\frac{1}{N}\sum_{f_{k}\in F}\frac{\sqrt{\left(R_{in}(f_{k},\theta_{k})-R_{g}{)}^{2}+X_{in}^{2}(f_{k},\theta_{k}\right)}}{R_{g}}$$

Based on the magnitude of X(k), the iterative process is divided into three stages: when 0.057≤X(k)<0.21, fast-convergence weights and a larger step size are adopted to prioritize reducing the overall impedance deviation; when 0.032≤X(k)<0.057, moderate weights and step sizes are utilized to balance the convergence rate and solution accuracy; and when 0≤X(k)<0.032, high-precision weights and a smaller step size are applied to enhance the matching accuracy within the target frequency band. Through this hierarchical adaptive strategy, the algorithm’s sensitivity to initial values is reduced, and the convergence stability under dual-band matching conditions is improved.

**Figure 5:**
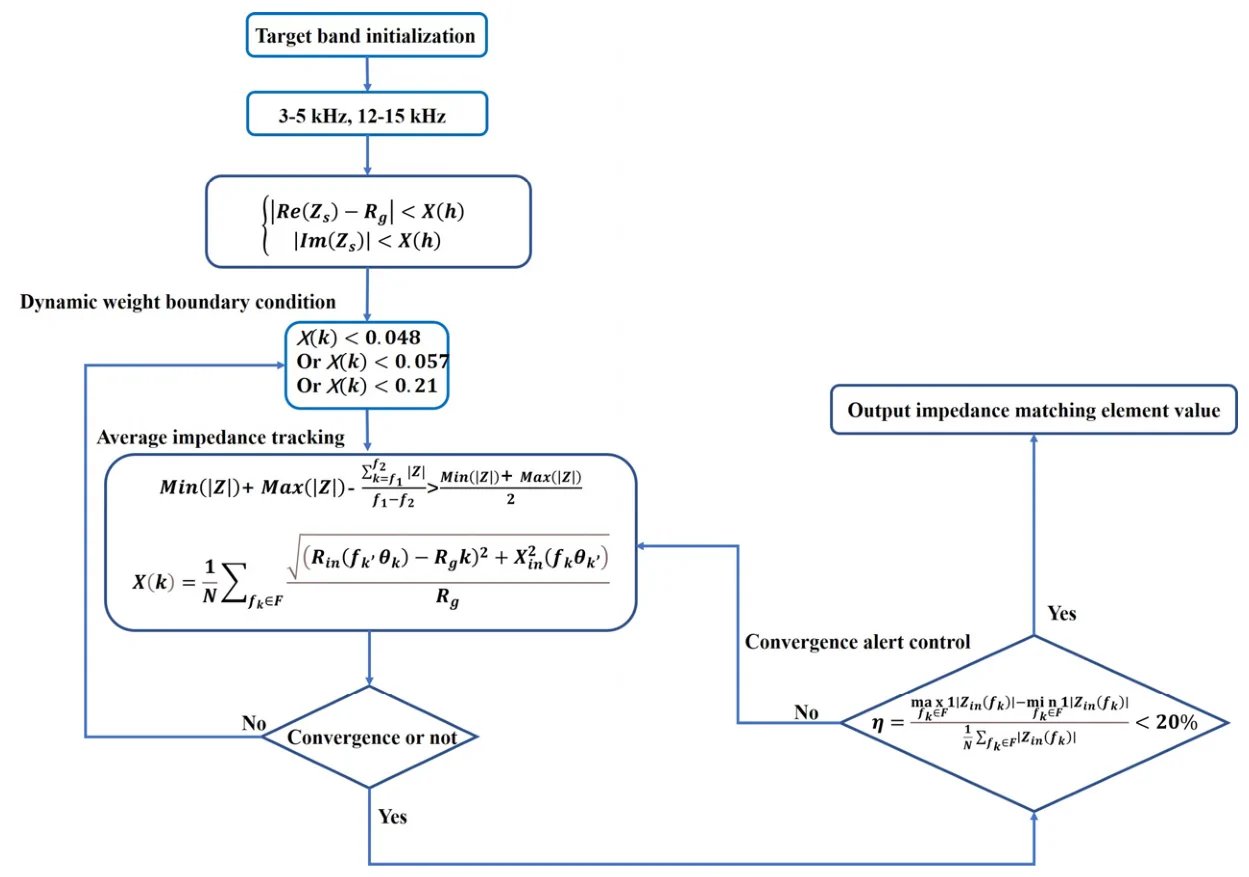
Flowchart of the adaptive Gauss–Newton algorithm for solving impedance matching network parameters.

Based on the aforementioned principles, the flowchart of the adaptive Gauss–Newton algorithm for solving the parameters of the broadband impedance matching network is illustrated in Figure 5. Initially, the target frequency bands are selected according to the logging requirements: the 3–5 kHz band for the quadrupole mode and the 12–15 kHz band for the monopole mode. The fundamental optimization objective is to make the real part of the input impedance within these bands approach the reference impedance, while the imaginary part approaches zero. Subsequently, the normalized impedance deviation is calculated based on the current iteration results, and the weights and step sizes are adaptively adjusted according to the predefined hierarchical thresholds. Following the weight update, the algorithm proceeds to the parameter iteration phase. The input impedances at discrete frequency points within the target bands are calculated based on the current parameters, and the matching network parameters are updated using the Gauss–Newton formula. In each iteration, the in-band impedance fluctuation coefficient is further calculated to evaluate the consistency of the impedance magnitude within the target bands. If the current parameters simultaneously satisfy both the impedance deviation constraint and the in-band fluctuation constraint, the algorithm is deemed to have converged; otherwise, it returns to the weight adjustment and parameter update phases for further optimization.

If the relative impedance fluctuation within the target frequency bands satisfies the predefined requirements, the component parameters of the impedance matching network are outputted; otherwise, the algorithm re-enters the iterative process to further refine the parameters. Through this comprehensive procedure, the requirements for real-part impedance matching, imaginary-part suppression, and in-band flatness are simultaneously satisfied within both target operating bands. Consequently, the matching network parameters suitable for the broadband excitation of LWD transducers are successfully obtained.

The impedance matching network parameters are shown in Table 3.

**Figure 6:**
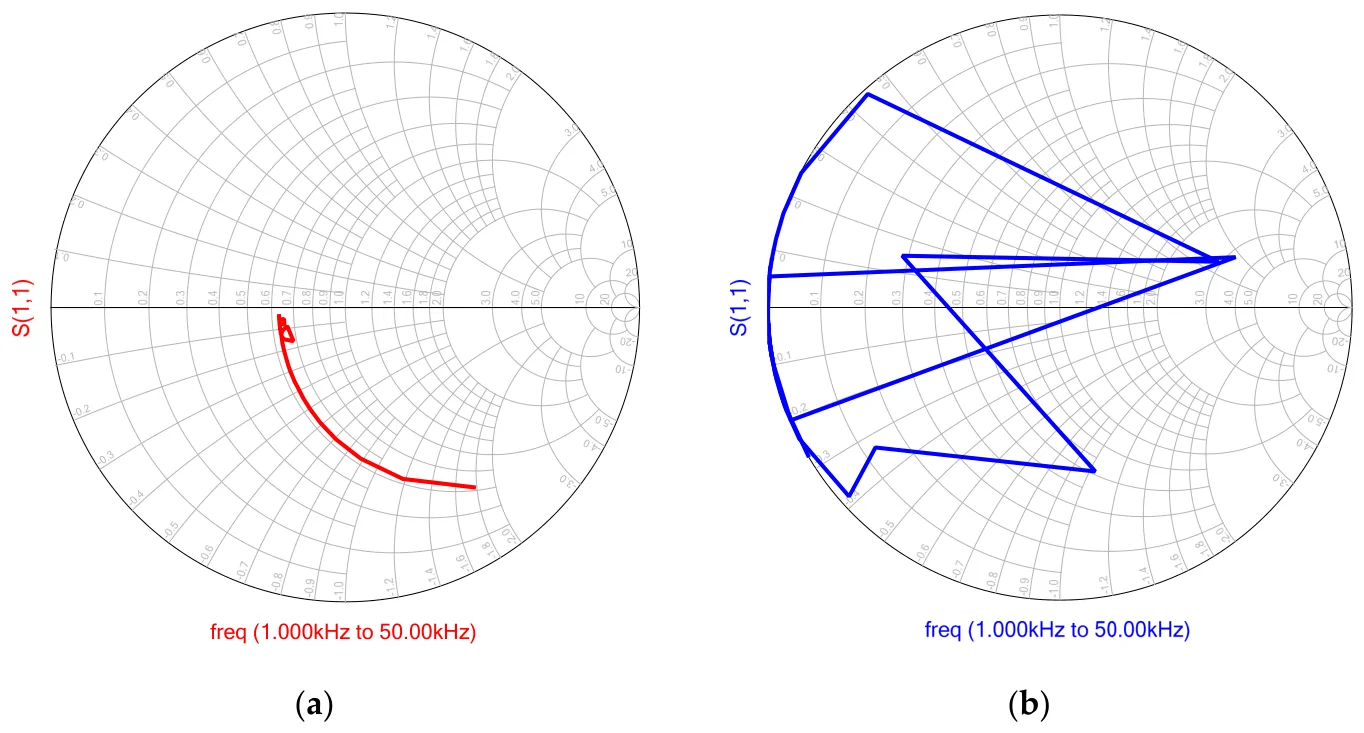
Comparison of reflection coefficient trajectories of transducer equivalent circuit ports. (**a**) Before impedance matching, (**b**) after impedance matching.

Simulations of the proposed impedance matching network and the transducer’s BVD equivalent circuit were conducted in ADS (Advanced Design System). Figure 6 shows that before matching, the reflection coefficient trajectory of the transducer port lies mainly on the right side of the Smith chart, far from the center, indicating a notable mismatch with the reference impedance. Because the transducer exhibits a strong capacitive load in the target band, its port impedance fluctuates significantly with frequency. This prevents the excitation source from efficiently delivering energy to the transducer; instead, part of the energy is reflected back to the front-end circuit, which restricts the active power delivered to the transducer.

To assess the practical robustness of the dual-band matching network, a tolerance sensitivity analysis was performed. Each of the six matching components was individually varied by ±5% and ±10%, corresponding to the typical tolerance range of commercial RF chip inductors and ceramic capacitors. The resulting center frequency shifts remain within ±1.5 kHz (<2% of the nominal center frequency), and the transmission magnitude variation is bounded within ±1.5 dB. The most sensitive element is $L_{3}$, whose ±10% variation causes ~±1.2 kHz shift; this can be mitigated by using a ±5%–tolerance inductor at modest additional cost. These results confirm that the design is practically realizable with standard off-the-shelf components.

Upon connecting the impedance matching network, the reflection coefficient trajectory on the Smith chart changes notably. The post-matching trajectory shifts toward the center matching region, demonstrating that the network achieves impedance transformation and reactive compensation. Consequently, the equivalent input impedance in the target band aligns better with the reference impedance. Despite some frequency-dependent fluctuations caused by the multimodal resonance of the LWD acoustic transducer, the overall matching condition is substantially improved.

Physically, the center of the Smith chart represents a low reflection coefficient and high matching degree, while the outer edge indicates strong energy reflection. Thus, the trajectory moving toward the center signifies reduced impedance mismatch between the excitation source and the transducer, enabling more efficient electrical energy transfer. This lays a theoretical foundation for the enhanced transducer voltage, power factor, and acoustic emission response observed in subsequent experiments.

## 4. Experimental Test Analysis

**Figure 7:**
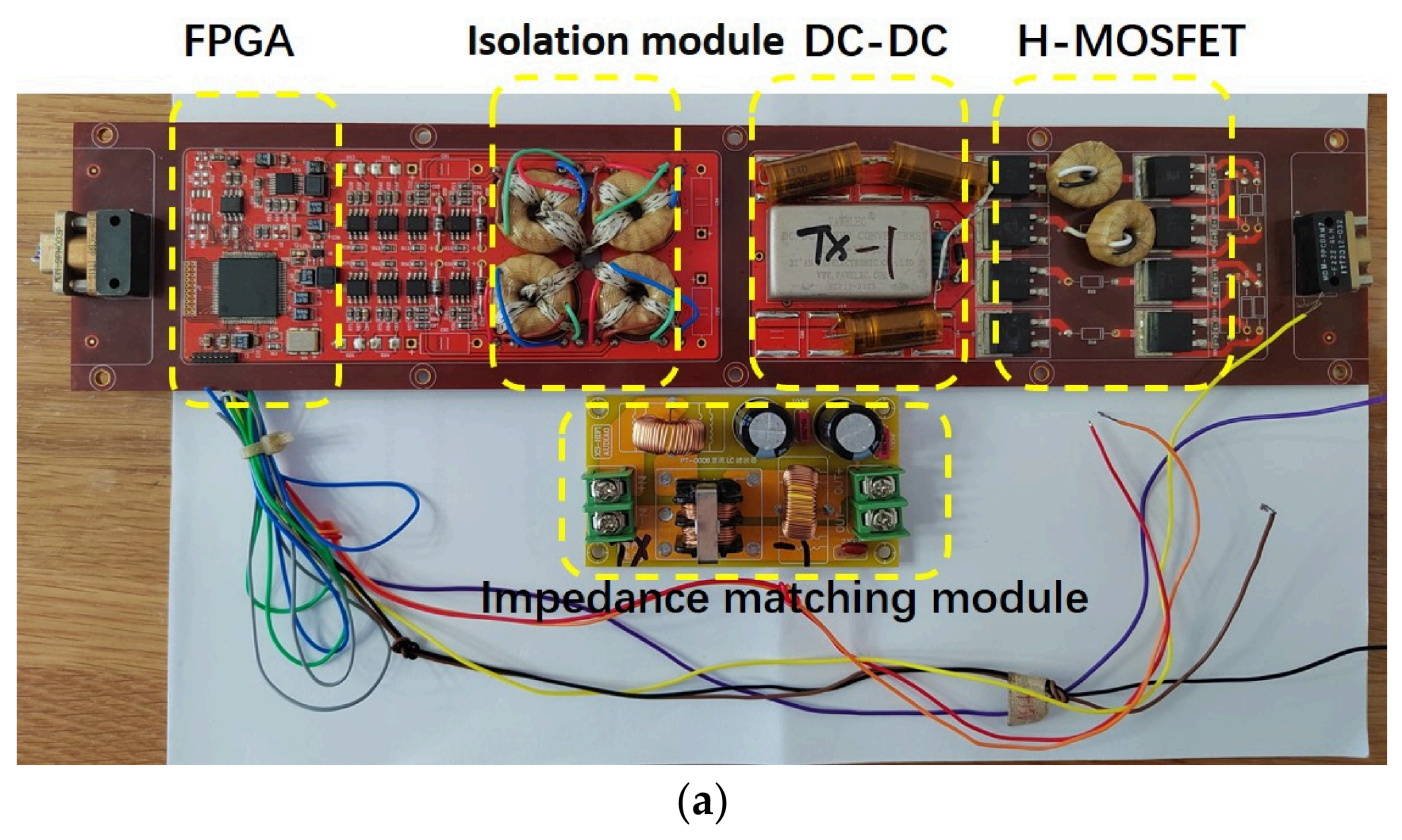

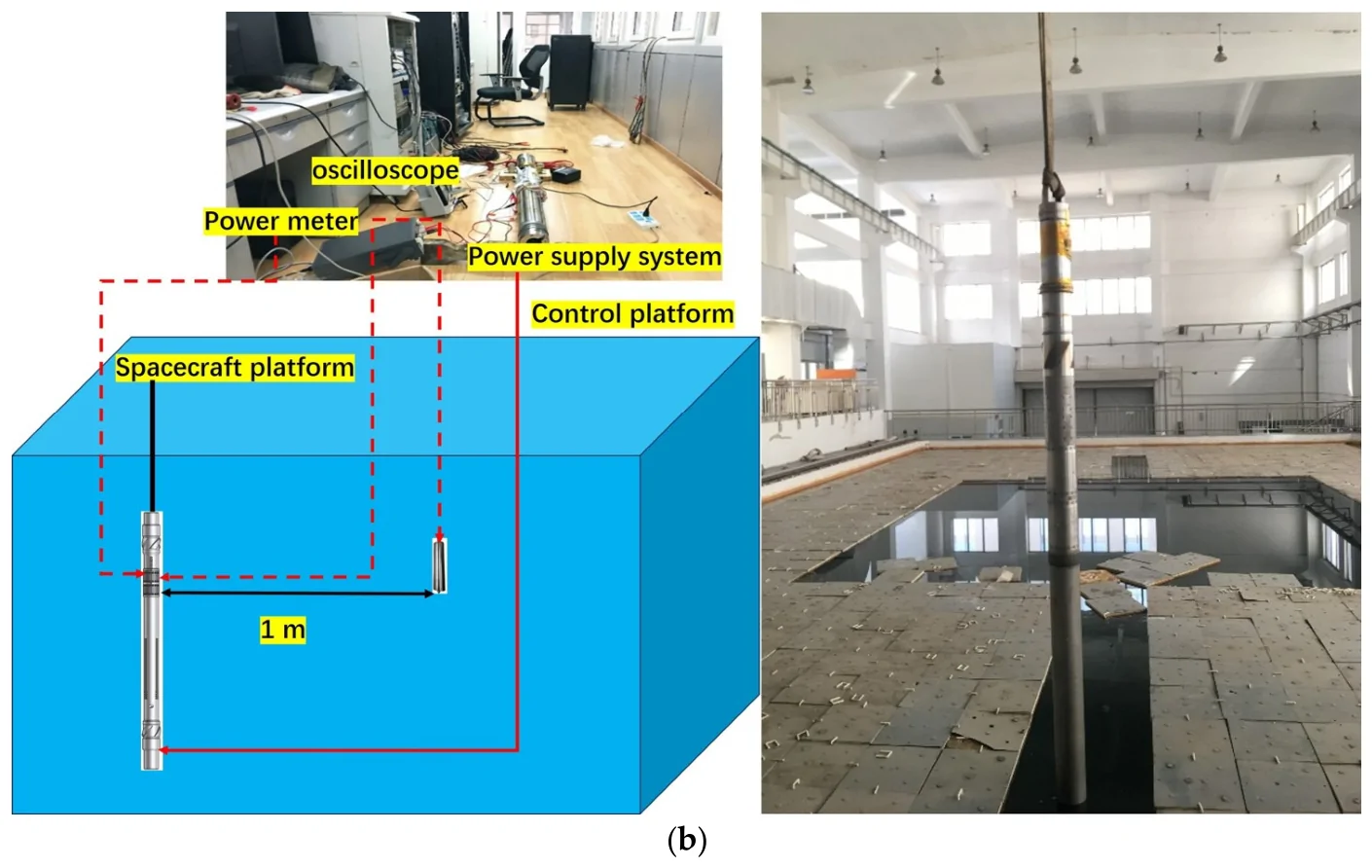
Experimental test environment. (**a**) Transducer excitation and signal acquisition system, (**b**) anechoic pool test.

The experimental setup is shown in Figure 7. Figure 7a illustrates the hardware circuit board of the broadband excitation system and the impedance matching module designed in this study. A separate design is adopted to facilitate replacement during subsequent experimental testing. The system utilizes an FPGA as the core control unit, which generates four timing-controllable drive signals to control a MOSFET full-bridge power switching circuit, thereby outputting high-voltage pulse signals. After passing through the impedance matching network and a step-up transformer, the excitation signal is applied across the transducer to achieve high-voltage excitation of the LWD acoustic transducer.

As shown in Figure 7b, after the individual modules of the instrument were fully assembled, the system was hoisted into an anechoic water tank for acoustic field experiments. The control console was used to suspend the instrument in the tank, with a hydrophone positioned at a distance of 1 m. The hydrophone converted the collected acoustic signals into electrical signals, which were then transmitted to an oscilloscope and the control console’s computer for data acquisition and processing. Based on this data, the sound pressure, acoustic radiation power, and horizontal directivity were calculated. Additionally, the control console was equipped with a power meter to measure the active power of the transducer. The hydrophone used in this paper is the Brüel & Kjær (B&K) 8104 hydrophone produced by the RESON Company of Denmark, Santa Barbara, CA, USA. The operating frequency range of the hydrophone is 1 hz~420 khz, and the receiving sensitivity is: −220 dB ref 1 V/μPa at 250 hz. All measurements were conducted in an anechoic water tank at room temperature (22 ± 1 °C), corresponding to a sound speed variation of less than 0.3%, which is negligible for our operating frequency. The tank was open to ambient atmosphere under hydrostatic pressure.

**Figure 8:**
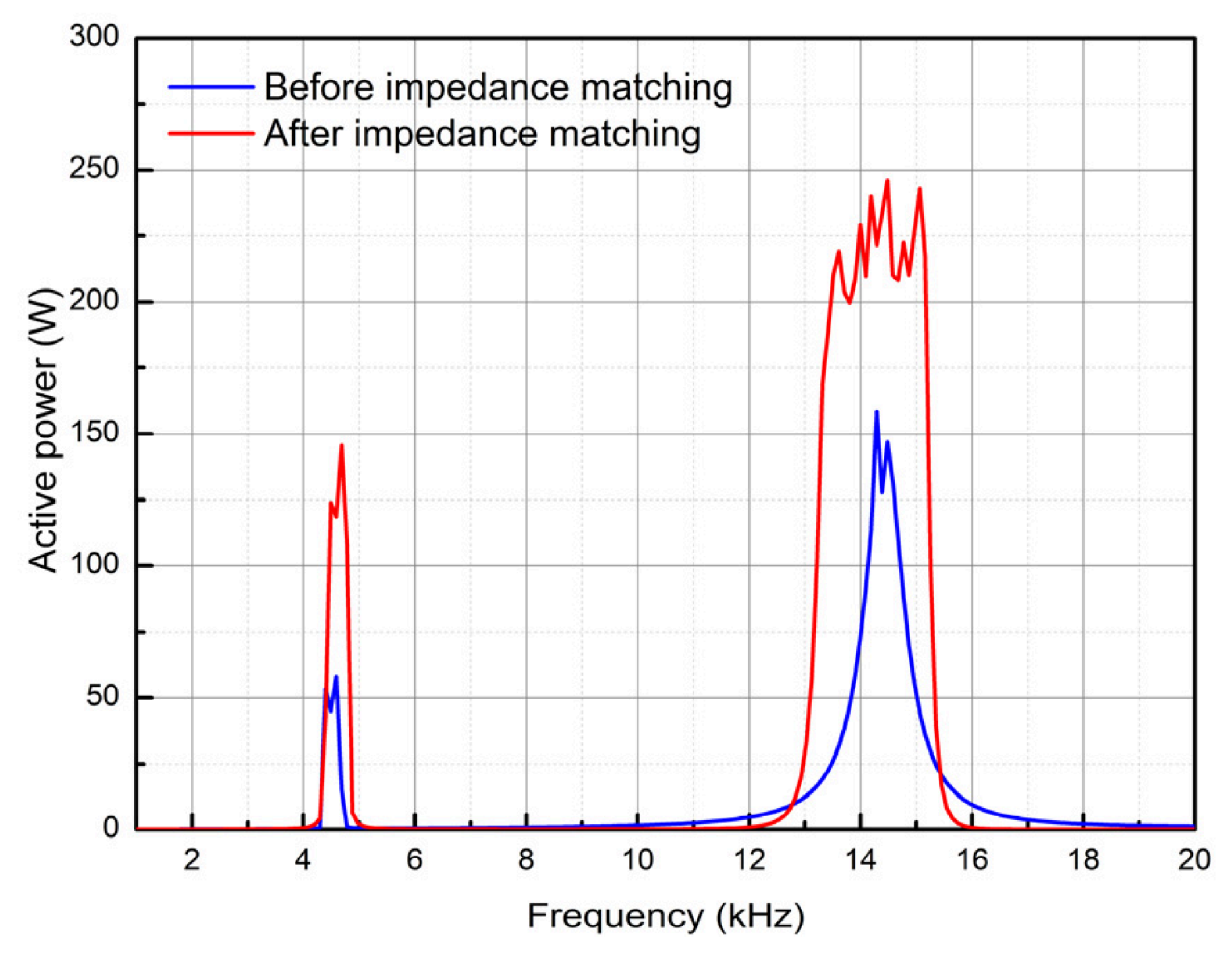
Active power before and after impedance matching.

As illustrated in Figure 8, prior to matching, the transducer’s active power is mainly concentrated around two narrow resonant frequencies. In the quadrupole band, the peak active power is about 50 W near 4.5 kHz, while in the monopole band, it reaches approximately 155 W near 14.3 kHz. The narrow pre-matching peaks indicate a limited effective energy input range in the target bands.

Upon connecting the impedance matching network, the active power in both target bands improves significantly. For the quadrupole mode near 4.5 kHz, the peak active power increases from ~50 W to ~148 W (a 2.9-fold enhancement). For the monopole mode in the 12–15 kHz band, the peak rises from ~155 W to ~248 W (a 1.7-fold increase). Furthermore, a broad high-power output range emerges in the monopole band, where the active power remains consistently high between 13.5 kHz and 15.2 kHz. This confirms that the matching network not only boosts the peak power but also improves the overall power transfer condition across the target frequency bands.

Table 4 shows that before matching, the transducer’s power factor is generally very low in both operating bands, dropping to the order of 0.01 near 4 kHz, 5 kHz, and 13.4–13.6 kHz, indicating strong reactivity. Upon connecting the matching network, the power factor improves significantly overall. In the quadrupole mode, the power factors at 4.6 kHz and 4.8 kHz increase from 0.118 and 0.163 to 0.52 and 0.478 (a 4.4-fold and 2.9-fold enhancement, respectively). However, the marginal improvement at the band-edge frequencies (4 kHz and 5 kHz) suggests that the network approaches its effective bandwidth limit at the edges. The monopole mode shows the most remarkable improvement: at 13.4 kHz and 13.6 kHz, where active power output was previously negligible, the power factors jump to 0.856 and 0.601. In the 13.8–14.4 kHz band, the values exceed 0.7, peaking at 0.972 at 14.4 kHz, approaching unity power factor. These results are achieved through optimal network parameters derived from the adaptive Gauss–Newton algorithm, which effectively compensates for the transducer’s static capacitance and reactive components in the target dual bands, significantly boosting the active power output.

**Figure 9:**
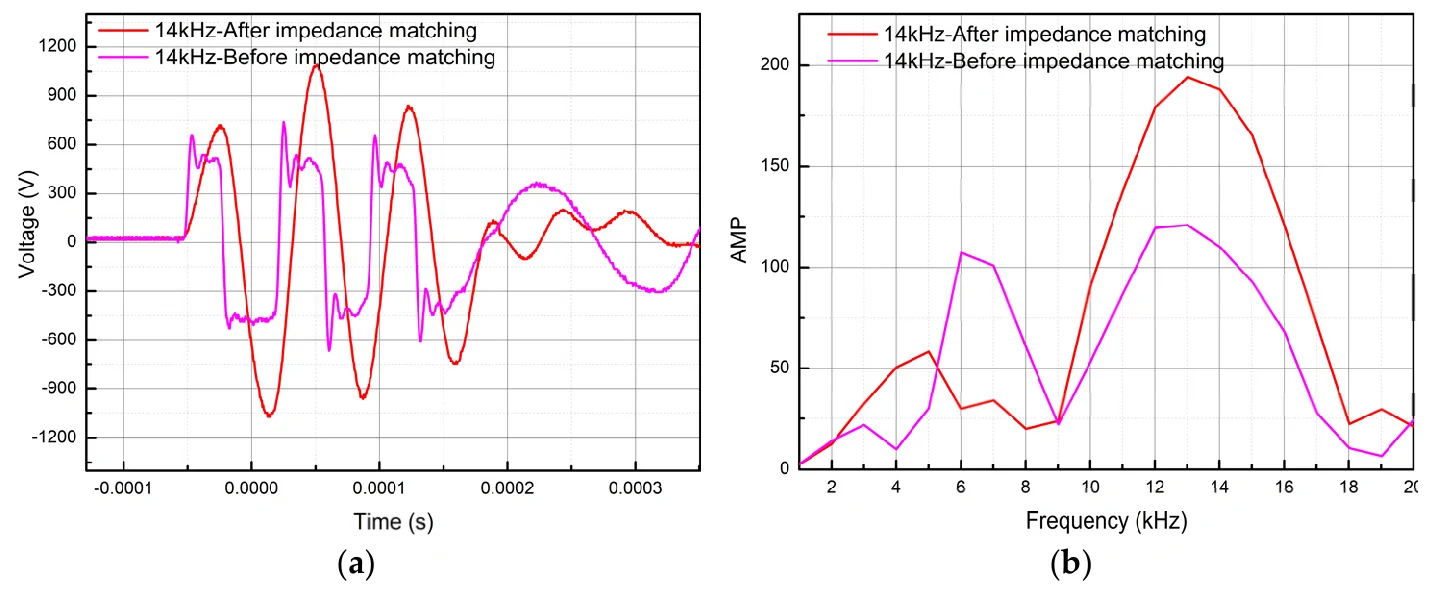
Transmitted Voltage Test for Monopole Mode. (**a**) 14 kHz emission voltage–time domain, (**b**) 14 kHz emission voltage–frequency domain.

Figure 9a shows that under 14 kHz excitation, the pre-matching transmitted voltage waveform at the transducer terminal displays obvious plateau and spike features, indicating the influence of the transducer’s capacitive load and downstream parasitic parameters. The pre-matching peak-to-peak voltage is ~1.2 kV. After impedance matching, the terminal voltage amplitude increases significantly to a peak-to-peak value of ~2.1 kV, which is about 1.75 times the pre-matching value. Furthermore, the waveform shifts from a pulsed plateau to a smoother periodic oscillation, indicating that the matching network suppresses high-frequency spikes and non-target frequencies.

Figure 9b reveals that before matching at 14 kHz, the frequency-domain amplitude of the transmitted signal contains notable non-target components near 6 kHz, in addition to the 12–15 kHz monopole target band. Post-matching, the amplitude within the 12–15 kHz band is significantly enhanced, reaching a maximum near 13 kHz. The main peak amplitude increases from 120 to 190 (a ~1.6-fold improvement). This confirms that the impedance matching network enhances the effective excitation within the monopole target band, concentrating the transmitted voltage spectrum in the desired operating frequency range.

**Figure 10:**
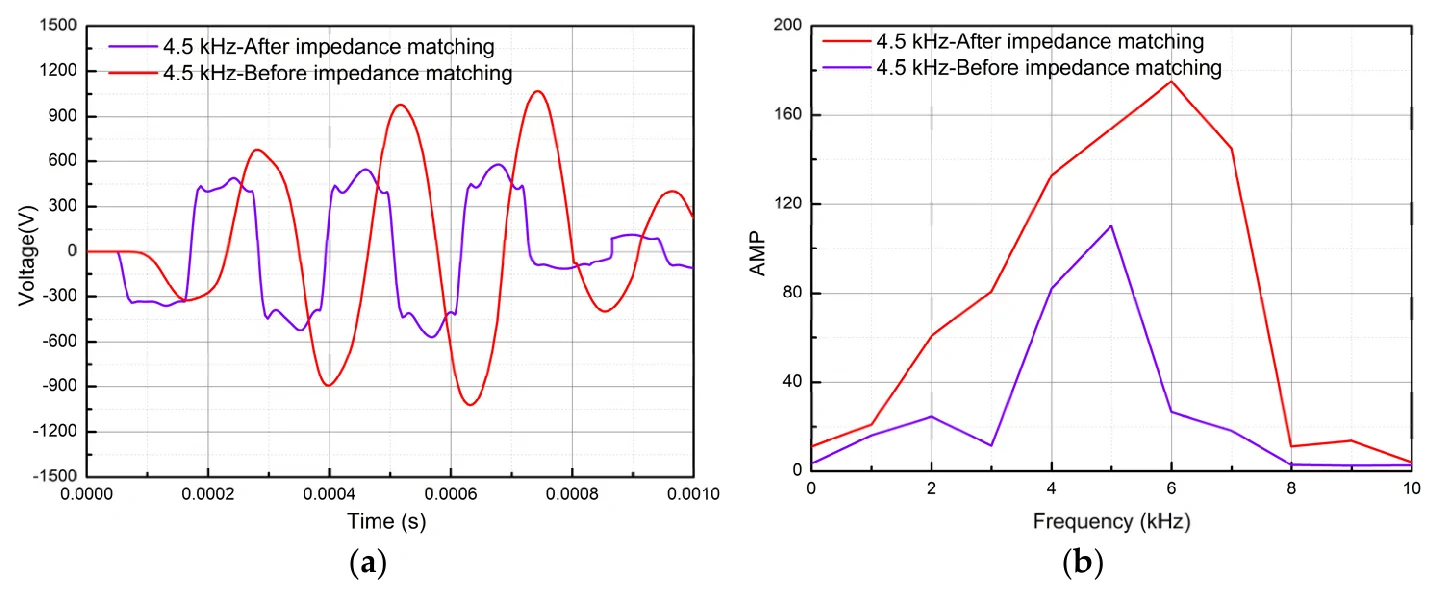
Transmitted Voltage Test for Quadrupole Mode. (**a**) 4.5 kHz emission voltage–time domain, (**b**) 4.5 kHz emission voltage–frequency domain.

Figure 10a shows that under 4.5 kHz excitation, the pre-matching transmitted voltage at the transducer terminal exhibits strong pulsed driving features with obvious waveform flattening and abrupt transitions, yielding a peak-to-peak voltage of ~1.2 kV. Post-matching, the positive and negative peaks reach ~1 kV and ~−1.1 kV, respectively, resulting in a peak-to-peak value of ~2.1 kV (an increase by a factor of ~1.75). Additionally, the waveform becomes significantly smoother, displaying distinct low-frequency periodic oscillations. This indicates that the matching network suppresses high-frequency harmonics in the original pulsed signal while boosting the voltage amplitude.

Figure 10b reveals that before matching at 4.5 kHz, the frequency-domain response is mainly concentrated near 4 kHz with a main peak amplitude of ~110. After matching, the amplitude within the target band increases significantly, peaking at ~175 near 5 kHz (a ~1.8-fold improvement). Moreover, a high amplitude is maintained across the 3–7 kHz range. This confirms that the impedance matching network not only enhances the transmitted voltage near the quadrupole target frequency but also broadens the effective excitation range within the low-frequency target band.

**Figure 11:**
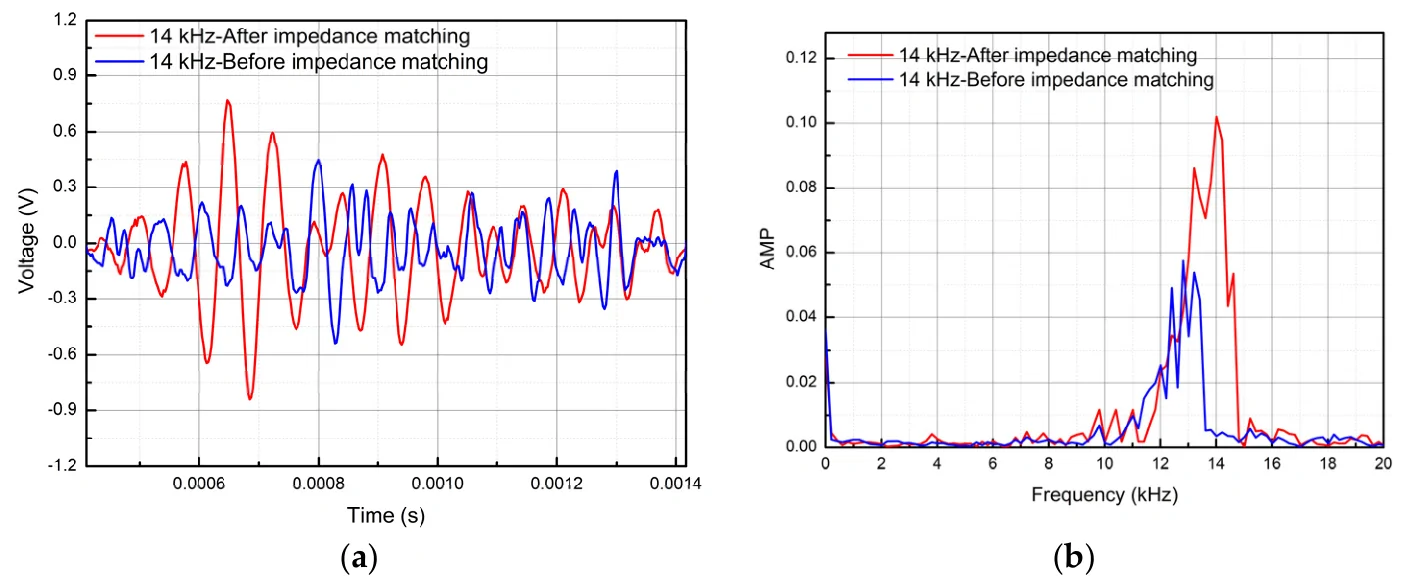
Received Voltage Test of Hydrophone. (**a**) 14 kHz hydrophone receiving voltage–time domain, (**b**) 14 kHz hydrophone receiving voltage–frequency domain.

Following the transmitted voltage tests, the hydrophone received voltage was further measured to evaluate the variation in the transducer’s acoustic radiation capability before and after impedance matching. Figure 11a shows that under 14 kHz excitation, the pre-matching peak-to-peak received voltage is ~1 V, which increases to ~1.9 V post-matching (a ~1.9-fold improvement). This indicates a significant enhancement in the acoustic radiation intensity at the monopole target frequency.

The frequency-domain analysis (Figure 11b) shows that the response is mainly concentrated within the 12–14.5 kHz range. Before matching, the main peak in the target band is near 13 kHz with an amplitude of ~0.055. After matching, the main peak shifts to ~14 kHz with an amplitude of ~0.11, representing a twofold increase. This demonstrates an enhanced acoustic radiation response within the monopole target band. Additionally, the overall frequency-domain amplitude in the 13–14.5 kHz range is significantly higher post-matching. These results confirm that the impedance matching network substantially improves both the response intensity near the target frequency and the effective acoustic radiation capability.

**Figure 12:**
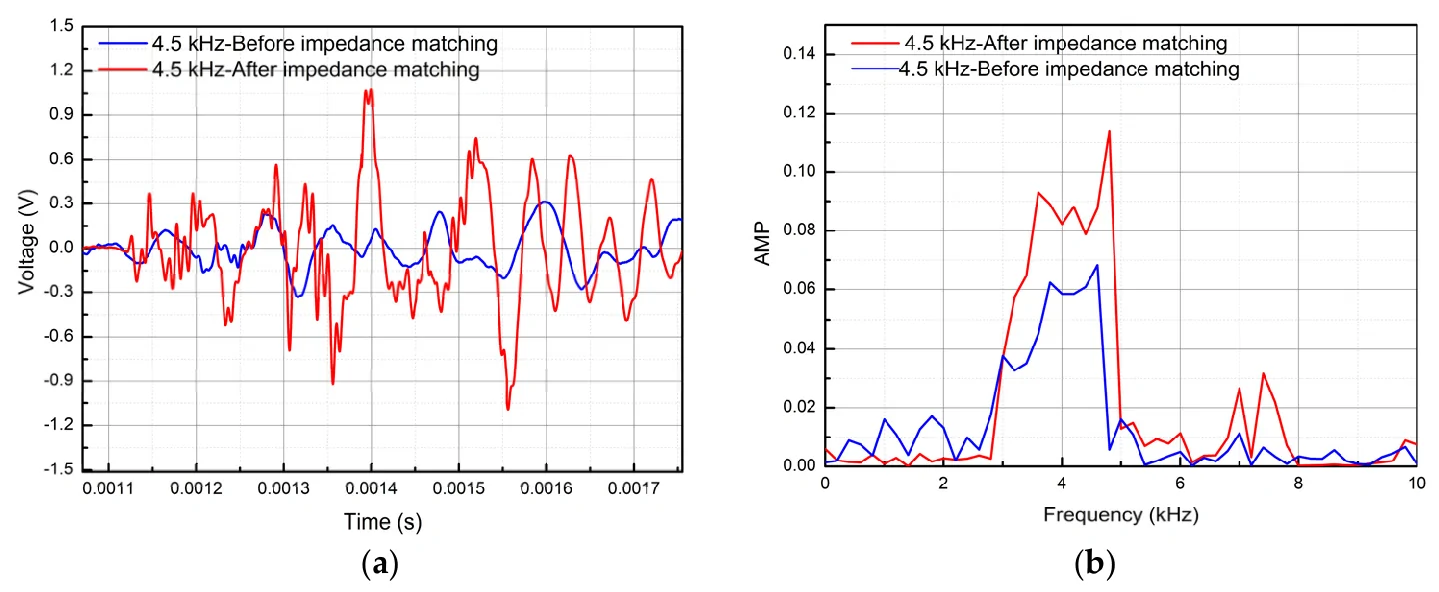
Received Voltage Test of Hydrophone. (**a**) 4.5 kHz hydrophone receiving voltage–time domain, (**b**) 4.5 kHz hydrophone receiving voltage–frequency domain.

Figure 12a shows that under 4.5 kHz excitation, the pre-matching hydrophone received voltage is relatively low (~0.65 V peak-to-peak). Post-matching, it increases significantly to ~2.35 V, representing a ~3.5-fold improvement. This indicates that the matching network substantially enhances the acoustic field output in the low-frequency quadrupole operating band.

The frequency-domain analysis (Figure 12b) reveals that the response is mainly distributed within the 3–5 kHz range. Before matching, the main peak amplitude near 4.5 kHz is ~0.08, which increases to ~0.11 post-matching (a ~1.5-fold improvement). Nevertheless, due to the inherently narrow bandwidth and low admittance of the transducer in the low-frequency quadrupole band, bandwidth broadening is highly challenging, leading to a limited expansion of the frequency band.

**Figure 13:**
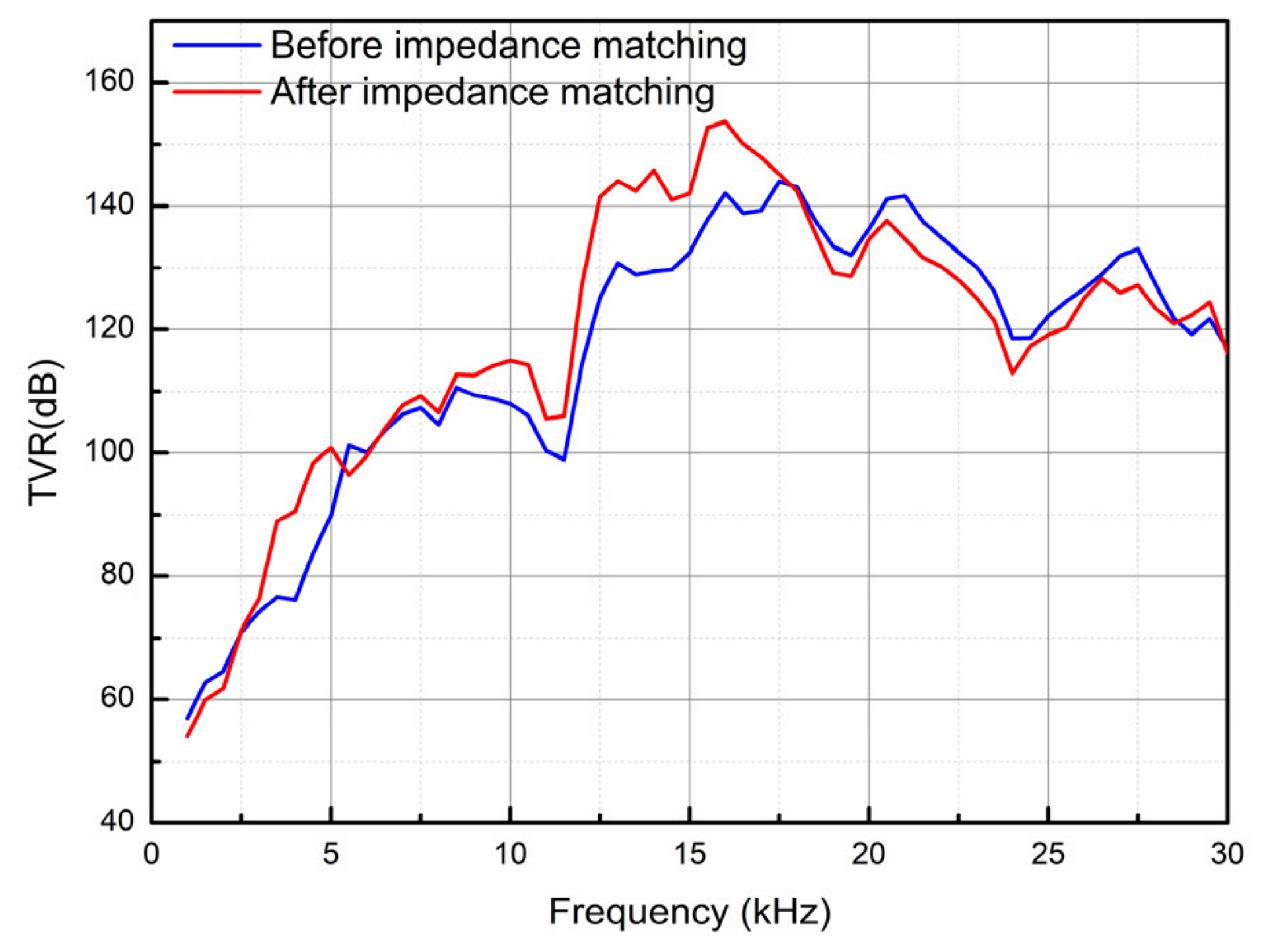
Transmitting Voltage Response Test.

To further evaluate the impact of the impedance matching network on the acoustic transmission capability of the transducer, the transmitting voltage response (TVR) before and after impedance matching was measured, with the results shown in Figure 13. After integrating the impedance matching network, the TVR significantly improves across the 9–16 kHz band. In the 12–15 kHz monopole target band, the TVR increases by 10–18 dB overall; notably, at 14 kHz, it rises by ~15 dB (from 130 dB to 145 dB), peaking near 150 dB at 15–16 kHz. This confirms the network effectively enhances acoustic transmission in the monopole band. In the 3–5 kHz quadrupole band, the TVR increases by 5–17 dB. However, bandwidth expansion remains limited due to the transducer’s inherently low admittance and bandwidth in this low-frequency range.

To further verify the spatial acoustic radiation characteristics before and after impedance matching, horizontal directivity measurements were conducted in an anechoic water tank. With a fixed distance between the transducer and the hydrophone in the same horizontal plane, the received signals were recorded across a 0–360° azimuthal range. The directivity patterns were then compared at the representative frequencies of 4.5 kHz (quadrupole mode) and 14 kHz (monopole mode). The measured normalized TVR curve is shown in Figure 13. The absolute peak TVR is approximately 162 μPa/V at the center frequency, with an estimated uncertainty of ±1.5 dB.

**Figure 14:**
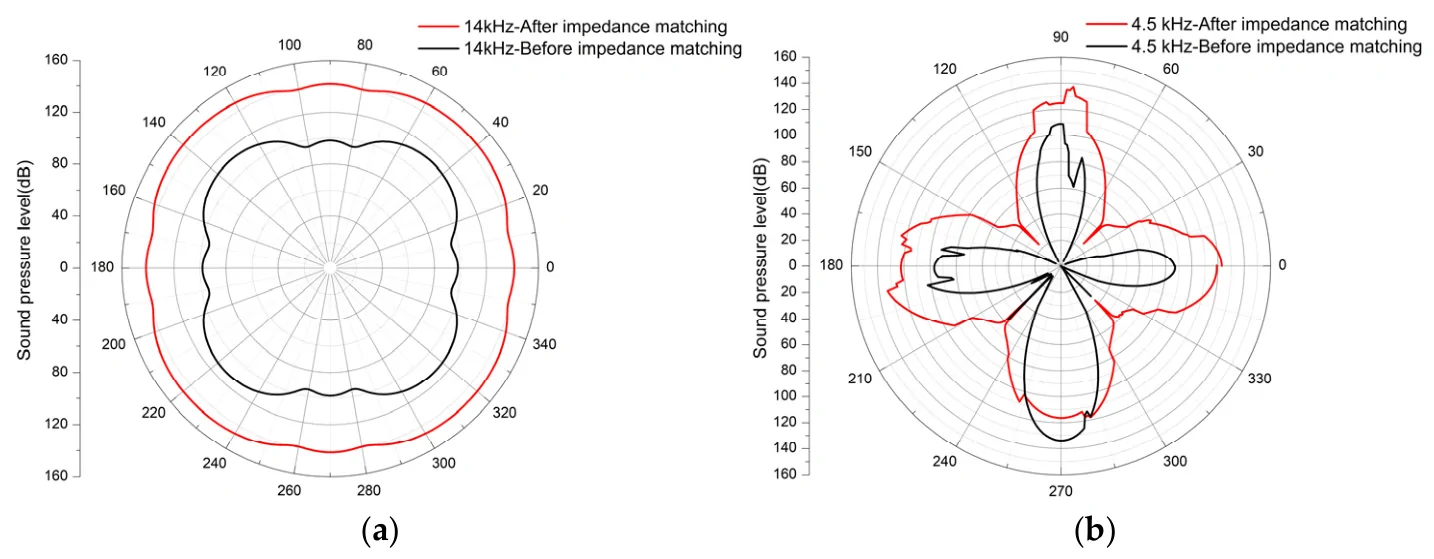
Horizontal directional testing. (**a**) Monopole mode—14 kHz, (**b**) Quadrupole mode—4.5 kHz.

Figure 14a demonstrates that at 14 kHz, the average SPL increases by 35 dB at 0°, 90°, 180°, and 270°. This confirms that the impedance matching network significantly boosts the monopole radiation intensity without severely degrading horizontal spatial uniformity. The uniform directivity gain across all directions makes the source more ideal compared to the unmatched state.

At 4.5 kHz (Figure 14b), the transducer initially shows weak quadrupole characteristics before matching. After applying the impedance matching network, a distinct four-leaf clover pattern with stronger directivity emerges. The directional gains are 16 dB (0°), 30 dB (90°), 22 dB (180°), and 56 dB (270°), averaging 28 dB. The pronounced gain at 270° is likely due to significant vibrational variations in the low-frequency mode, where underwater confining pressure may affect the output consistency.

Based on the above experimental tests, the sound field radiation characteristics of the novel transducer designed in this paper were higher than those of the conventional transducer. In addition, the working frequency band can be adjusted according to specific requirements, and the effectiveness of the impedance matching network was further verified through hydrophone testing.

## 5. Conclusions

To overcome the limitations of narrow bandwidth, significant capacitive loading, and low power transmission efficiency in logging-while-drilling (LWD) acoustic transmitters, this study proposes an FPGA-based broadband excitation method. Focusing on the monopole (12–15 kHz) and quadrupole (3–5 kHz) bands, an equivalent circuit model incorporating the mechanical coupling with the drill collar was developed to calculate the impedance matching network parameters. Experimental evaluations were then performed, yielding the following conclusions:

(1) Based on an anechoic water tank, systematic tests were performed to evaluate the electrical and acoustic performances of the LWD acoustic transducer before and after impedance matching. Results indicate a significant enhancement in electrical performance across both target frequency bands post-matching. Notably, the quadrupole mode shows the most substantial improvement, with active power reaching ~2.8 times the pre-matching value. In terms of acoustic performance, the monopole operating bandwidth expands from 14–14.8 kHz to 13.2–15.2 kHz (a 2.4-fold increase). Conversely, the quadrupole bandwidth expansion is limited, attributed to the transducer’s inherently low bandwidth and admittance in the low-frequency quadrupole band.
(2) The limited bandwidth expansion in the low-frequency band is primarily due to the transducer’s inherently low resonant admittance and high quality factor in the quadrupole excitation band, resulting in a significant bandwidth discrepancy with the monopole mode. Additionally, distinct vibration characteristics in the low-frequency mode cause non-uniform directivity enhancement under underwater confining pressure. Further research will be conducted on broadband excitation for low-frequency quadrupoles.
(3) In downhole environments, the transducer’s resonant frequency is prone to drift due to temperature and confining pressure. The existing circuit system lacks effective monitoring and feedback for the output voltage and current. Future work will focus on introducing an excitation signal control algorithm and a detection feedback circuit module to mitigate these issues.
(4) The present work focuses on the circuit design methodology and lab-scale validation under ambient conditions. For practical deployment, high-temperature-rated components (e.g., C0G ceramic capacitors, high-temperature inductors) would be required.

## Figures and Tables

**Table 1:** Transducer material parameters.

Section of the Transducer	Material	$\rho \;(kg/m^{3})$	c (m/s)	$d_{33}\;(C/m)$	$S_{ij}\;(N^{2}/m)$	Loss Factor
Inner drilling collar and Outside	Steel	7850	7850	-	-	0.002
Protective Cover	Steel	7850	7850	-	-	0.002
Piezo ceramic stack	PZT-5A	7750	2930	$39\times 10^{-11}$	$S_{33}^{E}=16.4\times 10^{-12}$ $S_{33}^{D}=82\times 10^{-13}$	0.01
Peek	Epoxy resin	1500	2740	-	-	0.004
cushioning material	Aluminum	2700	5800	-	-	0.003

**Table 2:** Transducer size parameters.

Section of the Transducer	Inner Diameter (mm)	Outer Diameter (mm)	Thickness (mm)	Height (mm)	Angular Span (deg)
Inner drilling collar and Outside	40	50	12	150	360
Protective Cover	60	62	2	120	90
Piezo ceramic stack	52	58	6	55	80
Peek	50	60	10	120	90
cushioning material	52	58	6	4	80

**Table 3:** Impedance matching network components.

Impedance Matching Component	Value
$C_{1}$	1.93 uF
$L_{1}$	91.41 uH
$L_{4}$	105.38 uH
$L_{2}$	180.26 uH
$C_{2}$	4.19 uF
$L_{3}$	420.82 uH

**Table 4:** Comparison of power factor before and after impedance matching.

Frequency (kHz)	Before Impedance Matching	After Impedance Matching
4.0	0.001	0.010
4.2	0.009	0.032
4.4	0.026	0.021
4.6	0.118	0.520
4.8	0.163	0.478
5.0	0.001	0.002
13.0	0.056	0.049
13.2	0.088	0.261
13.4	0.012	0.856
13.6	0.016	0.601
13.8	0.576	0.935
14	0.492	0.721
14.2	0.752	0.848
14.4	0.826	0.972
14.6	0.731	0.841
14.8	0.458	0.726
15	0.261	0.459

## Data Availability

The original contributions presented in this study are included in the article. Further inquiries can be directed to the corresponding authors.

## Appendix A

$$K_{1}=-\omega (\left(C_{2}\left(\omega^{2}L_{4}C_{1}+1\right)C_{2}+\omega^{2}C_{2}L_{1}-1\right)L_{3}-\omega^{2}L_{4}L_{2}L_{1}-L_{2}-L_{1})$$

$$K_{2}=\omega C_{2}\left(\omega L_{2}\left(\omega^{2}L_{4}L_{1}+1\right)-\omega L_{1}\right)+1+\omega^{2}L_{4}L_{1}$$

$$K_{3}=\omega^{2}(\left({-\omega }^{2}C_{2}\left(\omega^{2}C_{1}L_{4}L_{1}+C_{1}-L_{4}\right)L_{2}+C_{1}L_{1}\left(C_{2}-L_{4}\right)\omega^{2}-C_{2}\right)L_{T1}+\left(-\omega^{2}C_{1}L_{4}L_{1}-C_{1}{+L}_{4}\right)C_{2}$$

$$K_{4}={\left(\omega C_{1}R_{g}\right)}^{2}+{\left(\omega^{2}C_{1}L_{3}\right)}^{2},K_{5}={\left(\omega C_{2}R_{g}\right)}^{2}+{\left(\omega^{2}C_{2}L_{1}\right)}^{2},K_{6}={\left(\omega L_{4}R_{g}\right)}^{2}+{\left(\omega^{2}C_{1}L_{4}\right)}^{2}$$

$$K_{7}=\frac{\left(-\omega n^{2}\left(\left(\left(\omega^{2}C_{1}^{2}L_{1}-K_{4}C_{2}+C_{1}\right)K_{5}+K_{4}C_{2}\left(\omega^{2}C_{2}L_{2}+1\right)\right)K_{6}\right)\right)}{K_{4}K_{5}K_{6}}$$

$$K_{8}=\frac{\left(\left(K_{5}C_{1}^{2}R_{g}+K_{6}C_{2}^{2}\right)K_{4}+K_{5}K_{6}C_{1}^{2}\right)\omega^{2}n^{2}}{K_{4}K_{5}K_{6}}$$

$$K_{9}=\omega C_{2}\left(-\omega^{2}C_{1}L_{1}+\omega^{2}L_{2}\left(C_{1}-L_{4}\left(-\omega^{2}C_{1}L_{3}+1\right)\right)+1\right)+\omega (C_{1}{-L}_{4}\left(-\omega^{2}C_{1}L_{1}+1\right))$$

$$K_{10}={\left(\omega C_{1}R_{g}\right)}^{2}+{\left(\omega^{2}C_{2}L_{1}\right)}^{2},K_{11}={\left(\omega C_{2}R_{g}\right)}^{2}+{\left(\omega^{2}C_{2}L_{2}\right)}^{2},K_{12}={\left(\omega L_{4}R_{g}\right)}^{2}+{\left(\omega^{2}L_{4}C_{1}\right)}^{2}$$

$$K_{13}=\frac{K_{11}K_{12}\omega C_{0}-\frac{K_{11}}{K_{10}}K_{12}\left(\omega^{3}C_{1}^{2}L_{1}-\omega C_{1}\right)-K_{12}\left(\omega^{3}C_{2}^{2}L_{2}-\omega C_{2}\right)L_{3}))}{K_{11}K_{12}n^{-2}},K_{14}=\frac{n^{2}(\omega^{2}C_{1}^{2}R_{g}K_{11}K_{12}+\omega^{2}C_{2}^{2}R_{g}K_{10}K_{12}+\omega^{2}C_{3}^{2}R_{g}K_{10}K_{11})}{{K_{10}K}_{11}K_{12}}$$

$$K_{15}=(-K_{7}\left(K_{3}+1\right)K_{13}+K_{8}\left(K_{3}+1\right)K_{14}-K_{7}K_{9})$$

$$K_{16}=(K_{8}\left(K_{3}+1\right)K_{13}+K_{7}\left(K_{3}+1\right)K_{13}+K_{8}K_{9})$$

$$K_{17}=(\left(-K_{1}K_{8}+2K_{7}R_{g}\right)K_{13}-K_{14}\left(K_{1}K_{7}+2K_{8}R_{g}-K_{2}\right))$$

$$K_{18}=\left(K_{1}K_{7}+2K_{8}R_{g}-K_{2}\right)K_{13}-K_{14}(K_{1}K_{8}-2R_{g}K_{7})$$

The numerical stability of the $K_{i}$ expressions is verified through the following procedure:

Frequency sweep: All $K_{i}$ are evaluated over the frequency range 1–100 kHz at 1 Hz resolution. No poles, singularities, or sign reversals are observed outside the expected resonant frequencies. Perturbation test: Each matching component value is perturbed by ±5%, and the resulting variation in each $K_{i}$ remains bounded within ±7%, confirming that the expressions are well-conditioned with respect to component tolerances; Consistency check: The impedance computed via the full $K_{i}$ summation matches the direct numerical solution of Equations (28)–(30) to within machine precision ($10^{-12}$ relative error), validating the algebraic reduction.

## References

[1] Zhao Q., Che X., Qiao W., Tao S., Wang J. (2023). Inversion of radial formation velocity distribution based on monopole acoustic logging while drilling. Geoenergy Sci. Eng..

[2] Li Y., Wang Z., Su Y., Tang X. (2024). A theoretical analysis of the logging-while-drilling dipole acoustic reflection measurement. Front. Earth Sci..

[3] Wang J., Che X., Qiao W., Tao S., Zhao Q. (2025). Influence of the Transducer-Mounting Method on the Radiation Performance of Acoustic Sources Used in Monopole Acoustic Logging While Drilling. Sensors.

[4] Fu X., Gou Y. (2022). An analysis for unipole acoustic logging while drilling wavefields with varying eccentered collar positions. J. Geophys. Eng..

[5] Wang Q., Wang H., Ji Y., Wang J. (2025). A physics-constrained unsupervised machine-learning method for collar wave suppression in acoustic logging while drilling. Geophysics.

[6] Fu X., Gou Y. (2022). Novel transmitter transducer for acoustic logging while drilling. Appl. Acoust..

[7] Zhang K., Wang X., Tan B., Su Y., Li S. (2026). Machine learning in logging tools: Current applications, challenges, and future directions. J. Geophys. Eng..

[8] Weinzierl W., Schimschal S., Swiatek A., Geerits T. (2025). Geometrical optimization of an acoustic logging-while-drilling isolator. Proceedings of the SEG International Exposition and Annual Meeting, Houston, TX, USA, 25–28 August 2025.

[9] Sun Z., Tang X., He X., Liu X. (2020). Numerical simulations of wave fields for acoustic logging while drilling tools in a fluid-filled trough. J. Geophys. Eng..

[10] Shang H., Gao S. (2026). Characteristics of Reservoir Boundary Ranging with While-Drilling Impulse Sound Source. Sensors.

[11] Lu J., Men B., Che X. (2023). Experimental study on the receiving piezoelectric vibrator of azimuthal acoustic logging. J. Geophys. Eng..

[12] Al-Hemyari E., Pevzner R., Tertyshnikov K. (2026). Model-Driven Processing of Passive Seismic While Drilling Data Acquired Using Distributed Acoustic Sensing Without Conventional Drill-Bit Pilot Measurements. Sensors.

[13] Li Z., Shi Y., Li Y., Zhang W. (2025). Solving Dispersion Characteristics in Acoustic Logging While Drilling Instrumentation: Multi-Difference Algorithm. IEEE Transactions on Instrumentation and Measurement.

[14] Aeron S., Bose S., Valero H.P. (2015). Robust detection and estimation for logging while drilling monopole acoustic data. IEEE Trans. Signal Process..

[15] Li J.C., He X., Zhao A.S., Jiang C. (2023). Joint inversion of formation radial shear-velocity profiles by dipole acoustic logging while drilling. Geophysics.

[16] Ji Y., He X., Chen H., Wang X., Zhang H. (2021). Acoustic fields for monopole logging while drilling with an eccentric collar. Geophysics.

[17] Zhao Q.Q., Wang L., Qiao W.X., Che X.H., Liu C.D. (2026). Numerical Simulation of Azimuthal Acoustic Logging-While-Drilling Responses for Geosteering Applications. Appl. Geophys..

[18] Xu J., Hu H., Han B. (2024). A fast algorithm for simulation and analysis of wavefields in acoustic single-well imaging of logging-while-drilling considering arbitrary types of sources. Geophysics.

[19] Sorgini F., Mazzoni A., Massari L., Caliò R., Galassi C., Kukreja S.L., Sinibaldi E., Carrozza M.C., Oddo C.M. (2017). Encapsulation of piezoelectric transducers for sensory augmentation and substitution with wearable haptic devices. Micromachines.

[20] Jia L., Liang Y., Meng F., Wang Z., Zhang G., Wang R., Zhang W. (2024). Research on acoustic impedance matching method of CMUT sensor based on PDMS/BN particles. IEEE Sens. J..

[21] Zhou J., Bai J., Liu Y. (2022). Fabrication and modeling of matching system for air-coupled transducer. Micromachines.

[22] Huang H., Paramo D. (2011). Broadband electrical impedance matching for piezoelectric ultrasound transducers. IEEE Trans. Ultrason. Ferroelectr. Freq. Control.

[23] Yang X., Zhang Y., Zhang Z. (2024). A controllable T-type broadband impedance matching network for ultra-low frequency electroacoustic transducers. IEEE Trans. Power Electron..

[24] Yu J., Wang L., Tian W., Zhang K., Liao S., Xu Q. (2022). An adaptive broadband active impedance matching network for underwater electroacoustic transduction system. IEEE Trans. Ind. Electron..

[25] An J., Song K., Zhang S., Yang J., Cao P. (2014). Design of a Broadband Electrical Impedance Matching Network for Piezoelectric Ultrasound Transducers Based on a Genetic Algorithm. Sensors.

[26] Xin F., Yang G., Junfeng L. (2025). Broadband impedance matching design method for dipole acoustic logging transducer based on sparrow search algorithm. Sens. Actuators A Phys..

[27] Jeong Y., Lee K., Lee J., Lee C.H. (2026). Sequential Matching Circuit Design using Negative Impedance Converters for Wideband Power Transfer. IEEE Sens. J..

[28] Hu J., Lin S., Zhang X., Wang Y. (2014). Radially sandwiched composite transducers composed of the radially polarized piezoelectric ceramic circular ring and metal rings. Acta Acust. United Acust..

[29] Gou Y., Fu X., Wei F. (2023). Broadband Impedance Matching and Residual Vibration Attenuation for Underwater Acoustic Tonpilz Transducers Based on Adaptive Gauss Newton Algorithm. IEEE Trans. Instrum. Meas..

[30] Lickert F., Bruus H., Rossi M. (2022). Constant-Power versus Constant-Voltage Actuation in Frequency Sweeps for Acoustofluidic Applications. Micromachines.

[31] Lugo-Hernández E., Mirea T., Carmona J.M., Clement M., Olivares J., Collado J.C., Mateu J. (2023). Analysis of spurious peaks at series resonance in solidly mounted resonators by combined BVD-Mason modelling. Ultrasonics.

[32] Nastro A., Ferrari M., Rufer L., Basrour S., Ferrari V. (2022). Piezoelectric MEMS acoustic transducer with electrically-tunable resonant frequency. Micromachines.

[33] Yang X., Zhang Z., Xu M., Li S., Zhang Y., Zhu X.-F., Ouyang X., Alù A. (2024). Digital non-Foster-inspired electronics for broadband impedance matching. Nat. Commun..

